# Supplementary material for: Audio, video, chat, email, or survey: How much does online interview mode matter?
Source: PLoS One. 2022 Feb 22;17(2):e0263876. doi: 10.1371/journal.pone.0263876 (PMC8863281; doi:10.1371/journal.pone.0263876)
Supplement: S1 Protocol — Handbook containing interviewer instructions, interview script, and procedures. (PDF) [file pone.0263876.s001.pdf]

Summer 2019

Interviewer Handbook

Interviewer Handbook **0**

Overview **1**

Purpose of this study 1

Websites 2

Expectations **2**

Study Tasks 2

Training Session 3

Scheduling Interviews 3

Updating Calendar 3

Conducting Interviews 3

Check-in Meetings 4

Communication 4

Data & Confidentiality 4

Compensation 5

Interview Invitations **5**

Update Calendly 5

Interview Invites & Scheduling 6

Conducting Interviews **7**

General Guidance 7

Probes 8

Video 9

Audio 10

Email 11

Chat 13

Anonymous Chat 14

Scheduled Survey 15

Survey 15

What to do if… **16**

Interviewee is late/no-show 16

I am late/no-show 16

Need to reschedule/cancel 16

Audio or video isn’t working 16

Other technical difficulties 17

Interviewee was inappropriate/I feel unsafe 17

Interviewee has strong negative response 17

Appendix **18**

Configuring Calendly 18

Email Templates 19

Video/Audio/Chat Invite 19

Email Invite 20

Scheduled Survey Invite 21

Survey Invite 22

Interviewee-late 23

Interviewee-no-show 24

Interviewer-no-show 24

Example Email Interview 24

Copy of Your Consent Form 24

Overview

Purpose of this study

Interviews are a great way to generate rich data about people’s nuanced lives. As a qualitative method, interviewing can capture a moment in time for a person’s perspective on their stories, experiences, memories, and attitude. However, interviews are not necessarily the best method for collecting data across time. They usually ask interviewees to remember stories or experiences on-the-spot, so they can be affected by recency bias or retell an experience in a different depending on spurious factors like their current emotional state. If I ask you to tell me about your childhood in an interview, your answers may vary drastically depending on whether you got in a fight with your mother yesterday. To better

understand *how* people reflect on and reason about their experiences, you'll be asking a series of interview questions designed to spark personal reflection.

Some example relevant exploratory research questions:

- If asked to recall a moment in time, from which eras of life do people tend to recall memories?
- When considering personal development, how much change in themselves to people perceive?
- How much do people practice self-distancing during interviews?

During interviews, we'll ask you to gently guide the interviewees into focusing on personal growth and specific memories in time. We're conducting around 150 interviews to gather a wide variety of stories and perspectives across the US. Thanks for your help! We ask that you try to refrain from reading too much literature on this topic or forming hypotheses to these research questions; part of the intent behind exploratory studies is to come in with an open mind. We need your help to collect organic data. Along the way, we'll be trying out a variety of interview styles in order to test and improve online interview platforms and processes.

Websites

Since you already use CMU applications, it may be easier for you if you log in to your study accounts with 'private browsing' or on a separate browser.

Calendly - https://calendly.com/login (password is *not* the CMU password)

Email - email.cmu.edu (password is your cylab-interviewer password,

Google Drive - drive.google.com

Expectations

Study Tasks

There are several recurring tasks that we expect participants to complete to remain enrolled in the study. Detailed instructions for tasks are given in other sections of the handbook. If you ever cannot complete a study task on its normal recurring schedule, please notify us in advance and we can work together to accommodate that.

Task	Estimated Time	Frequency	Compensation
Training Session	5 hours	once	$60
Schedule interviews	<5 min	around daily	-
Update calendar	-	ongoing	-
Complete interview &	15-60 min	58-60 over 4 weeks	$12

follow-up questions			
Check-in meeting	30-60 min	4-5 meetings, weekly	$12

Training Session

If you're reading this, you've probably already attended the training session. The session is around 5 hours long, and will cover both interview best-practices and study logistics.

Scheduling Interviews

Approximately daily, you will receive a list of new interviewees that have been assigned to you. You are responsible for sending them an email inviting them to interview with you. If an interviewee cannot attend any of the scheduled time slots you have available or needs to reschedule an interview, you are responsible for coordinating with them to find another time.

Updating Calendar

To schedule interviews, we are using a website, Calendly, that allows interviewees to select an appointment slot from you calendar. Please log in to this site periodically to ensure your calendar is accurate. If you're logging in for the first time, follow the appendix on configuring your calendar. You are welcome to set your own hours, provided that you can set aside at least 20 hours/week for interviewees to choose from.

Conducting Interviews

The bulk of the study's time is spent conducting interviews. To respect the time of our interviewees, you are expected to conduct interviews on time, with the interview "room" set up and materials prepared at least 5 minutes before the start of the interview. Missing more than one interview is grounds for being removed from the study.

As you are completing many interviews, you may experience disinterest with the task. We expect that you nonetheless give interviewees respect by engaging with them as though you were indeed interested, by acknowledging and affirming their responses, using positive body language, and replying to their responses in a timely and relevant manner.

After each interview, you are expected to complete a short questionnaire about how the interview went. This questionnaire *will not* be used as an evaluation of your skill or study eligibility; completing it honestly is the only requirement. We anticipate that each interview will take at most 1 hour, including scheduling, set up, interview, and follow-up time. Interviews will be conducted via instant messaging, video, audio, email, or a survey interview, depending on the interviewee's preference and study needs.

Survey interviews are the quickest & least involved; you'll invite the interviewee to take a survey version of the interview instead of interviewing with you, which shouldn't take much of your time.

Audio, video, and **chat interviews** will use approximately 30 minutes of interviewing with 15 additional minutes for you and the interviewee to complete follow-up questionnaires. Plan for an hour block of time for these, as things sometimes run longer.

Email interviews are a little unusual because they are asynchronous. The ~10-minute kickoff of the interview will be scheduled, where you'll share some introductory content. After the interview questions actually begin, you and the interviewee are welcome to reply at your leisure. You should reply to email interview responses within 12 hours, though preferably in less time than that. We expect you to spend around 30 minutes cumulatively reading and responding to interview emails.

Check-in Meetings

Approximately weekly, you are expected to attend a check-in meeting in-person on CMU's campus. These meetings are scheduled in advance for 1 hour, we anticipate they will usually be shorter. You are responsible for any transportation costs to attend meetings. These meetings will give us the opportunity to confirm your completion of study tasks, discuss any problems or insights you've encountered during interviews, and occasionally learn about a topic in interviews methodology.

Communication

We expect study participants to respond to email communication promptly, responding within one day. We anticipate sending an approximately daily update during the evening that contains information about new interviewees and a few reminders for you. Being under-communicative is grounds for being removed from the study.

If you make a mistake (e.g., accidentally miss an interview) or something unusual happens (e.g., the scheduling website is down), we expect that you will promptly notify us. With so many interviews, we'll inevitably all make some mistakes; the more important thing is to keep everyone informed so we can identify and mitigate problems.

Data & Confidentiality

The people you interview are research subjects, protected by university policy and federal guidelines. It is crucial that we respect their confidentiality and preserve their anonymity as much as possible.

Interviewee Confidentiality: The responses and stories you hear during interviews may be unusual or interesting, but they are not our stories to tell. Please resist the urge to share interviewee responses with others, even if the stories seem anonymous. It can be really tempting to want to talk to someone; if you have this urge, feel free to reach out via email or use part of the check-in meeting to get any compelling or troubling interviewee responses off your chest. Likewise, please do not make copies of or screenshot interview responses.

Data Management: All data about interviewees, including email address, Prolific ID, interview responses, and email correspondence, needs to stay on university-controlled accounts and servers. Do not download or copy any of that information. For example, please do not sync your interview emails to your phone or personal device; they should be accessed only through your browser. If you take any notes

related to your interview, that data needs to stay in Google Drive. At the final check-in meeting, we will work together to ensure no interviewee data is stored on your devices.

Your interview, Calendly, and Zoom accounts should be used *only* for study purposes. Emails, documents, and scheduling pertaining to interviews should be completed exclusively using your study account.

Security: Please ensure that the password you selected for you interview account is not reused on any other accounts. We strongly recommend downloading and using a password manager to track and maintain strong passwords. For the duration of the study, please make sure to physically and digitally secure your device. If you have monitoring software such as parental control software that observes your device, please let us know. If you ever suspect that your device is infected with malware, or any study data becomes compromised, notify us immediately so that we can take steps to mitigate possible information breaches.

You should always use a WiFi network you control or trust; avoid coffeeshops. If you must use an unknown network or are unsure about network security, always configure and use the CMU VPN during interviews.

Your Safety: There are many steps you can take to protect your identity and safety during interviews. Feel free to choose a pseudonym to use for interviews if you'd prefer. We're providing a dedicated email address to use for this study to protect your personal information. While these interviews may make you feel embarrassed or uncomfortable, they are not intended to facilitate harm or harassment. If you ever feel unsafe or harassed during an interview or correspondence, we encourage you to immediately terminate the interview and reach out about the experience.

Compensation

Compensation will be combined every 2 weeks from the start of the training session, accruing according to what tasks you have completed. There may be a1-3 week delay until the payment is disbursed. We will notify you as soon as your compensation is available to be collected. A check will either be mailed to the address you provide, or you can request to pick up a check or cash at the CMU office. If you are already on CMU payroll & already have direct deposit set up, you may also be able to receive payments that way. Please ask if you have questions or preferences about any of these options!

You don't accrue compensation for no-show interviewees. We may revisit this policy if the number of no-shows becomes unreasonable.

Interview Invitations

Update Calendly

Every few days, or whenever relevant, make sure to update your calendar in Calendly. You should especially do this before you invite new interviewees to schedule with you, to make sure they'll be

signing up for times you're actually available! To update all or most of your interview types with the same schedule, follow the bulk edit instructions on the Calendly site. If you only want to update *one* event type (e.g., you only want to do email interviews on Mondays), do the following:

- Log in to Calendly and go to the "Event Type" menu under your account (https://calendly.com/event_types/user/me).
- Select one of the 5 interview events and click edit under the settings drop down.

[Image: screenshot of interface showing interviewer how to edit one of their interview events]
Image removed for licensing purposes.

- Select the "When can people book this event?" tab and scroll down to the calendar. Update your availability for all of the time slots that you are available to conduct an interview.

[Image: screenshot of interface showing interviewers where the button is to update their availability]
Image removed for licensing purposes.

- Once complete, click the blue "Save & Close" button and then go back to the "Event Types" menu. For each of the other 4 event types, edit them just as before and go to the "When can people book this event?" tab. Instead of manually entering your availability again, select the "Copy availability from…" option in the top right of the calendar and select the event which you have previously updated. Repeat for all remaining event types.

[Image: screenshot showing interviewers how they could copy their availability across events]
Image removed for licensing purposes.

Interview Invites & Scheduling

Approximately daily, we will email you a list of new interviewees with an interview type to invite and schedule. Information about interviewees will be stored in your account's Google Drive "Interviewee Tracking" spreadsheet. You are responsible for inviting them to interview with you.

The scheduling link for each type of interview will be different, so please set up your invitations carefully. Please note that the [X] in the template links must be replaced with your interviewer number. See email templates for full details.

Here's what the usual flow will look like:

1. Upon email from us, you have new assigned interviewees
2. Update your Calendly calendars
3. Open both your Google Drive “Interviewee Tracking” spreadsheet and your study Gmail
4. For each new interviewee:
    a. Check the interview type on the spreadsheet
    b. Create a new Gmail message from the corresponding email template using canned responses
    c. Fill in any missing information in the template
    d. Attach a copy of the interviewee consent form (found in your Drive)
    e. In your spreadsheet, change status to “Invited”
5. Wait for the interviewee to sign up. They will:
    a. Receive an invitation email
    b. Click the Calendly link
    c. Select a date and time from the list of available time slots

[Image: screenshot of calendar interface interviewees will see when signing up for an interview]

Image removed for licensing purposes.

6. Both you and the interviewee will receive a confirmation email
    a. An event will be added to your calendar.
    b. Interviewee will also receive an automatic reminder email 24 hours and 1 hour in advance with instructions for accessing the interview platform
    c. In your spreadsheet, change status to “Scheduled”
7. At least five minutes before the scheduled interview, set up your interview.

Conducting Interviews

General Guidance

The details of interview protocols will be slightly different depending on the type of interview. Make sure you follow the appropriate protocol and script. Begin at least 5 minutes before the interview. Open your email inbox, interview handbook, and tracking spreadsheet.

The script for your interview is not set in stone; feel free to adapt it to suit your version of a friendly but professional tone. However, in general, the interview script needs to follow this outline:

- Introduce yourself. Notify of recording audio/video.
- Clarify any questions on consent form
- Expectations

  - Around 10 questions over the next 30 or so minutes.
  - No wrong answers and everything you say will be kept confidential, so just try to answer honestly.
  - Avoid sharing identifying information about yourself or others
  - Free to skip questions
- Questions (~25 min)
  - What are your favorite things to do in your free time?
  - What characteristics of yourself are you most proud of?
  - What are your feelings and attitudes about death?
  - What has been the biggest disappointment in your life?
  - What is your most common sexual fantasy?
  - What have you done in your life that you feel most guilty about?
  - What characteristics of your best friend really bother you?
- Anything else to add?
- Share survey link (specific to interview type)
- Compensation information. $10 as a bonus on Prolific within 48 hours.
- Contact information interview-study@andrew.cmu.edu.

Try to keep an eye on the time and pace your questions to aim for the 30-minute mark. Try to get through all the questions while maintaining quality. If both you and the interviewee are engaged and willing, you're welcome to continue the interview for up to an hour. (People can be very interesting!)

Probes

An important part of interviewing is gently guiding the interviewee to generate data that is relevant to your research questions. The follow-up questions you ask after each question are crucial for encouraging rich data. Always be thinking about how you can guide the interviewee to help answer our research goals, without unduly influencing them. Here's some example follow-ups:

- Could you elaborate more on that?
- Say more about X? :)
- Have you always [felt like X], or was something that's developed over time?
- Have you always [felt that way about X], or was there a moment that changed things for you?
- Was there ever a specific moment that solidified that [feeling] for you?
- Can you think of a specific memory that illustrates that point?
- What do you mean by "X"?
- If you could go back and revisit, would you change anything about that moment?
- How do you feel about the fact that X?
- That makes sense to me.
- I'm not sure I follow.
- [audio, video] Mhm? Oh?

In general, it's good practice to mirror the language phrasing and style that your interviewee is using, even if it's not exactly what you would normally use. If an interviewee uses emojis or casual language, use those as well. If they capitalize and punctuate every sentence, follow their lead. If they call their childhood a "shitshow," your might quote that language in your follow-up questions (e.g., "Have you always considered your childhood a shitshow, or did that perspective change over time?").

Video

Tips: Ensure that your environment does not have personal information or distractions in the background. Because video can require troubleshooting, set up early and test your setup. Be aware of the tone that is set by things like the lighting, your body language, and your eye contact.

Set Up: Start the zoom meeting by clicking the link in your calendar event. Join with computer audio. Enable your audio and video. Help troubleshoot their issues.

Interview Script

- Hi! Thanks for joining me for this interview. I'm [name]. As a reminder, this interview is being recorded.
- So you agreed to a consent form earlier. I just wanted to check whether you have any questions about that before we begin?
- Great! I'm going to ask you around 10 questions over the next 30 or so minutes. There's no wrong answers and everything you say will be kept confidential, so just try to answer honestly. Try to avoid sharing identifying information about yourself or others; I'm on a need-to-know basis. For example, feel free to use fake names or just an initial when telling stories about others.
- If there's ever a question you're not comfortable with or don't wish to answer, feel free to say so and we can just move on instead. Sound good?

1. And just as a warning, It might seem like the questions jump around, so don't be taken off guard. :) What are your favorite things to do in your free time?
2. What characteristics of yourself are you most proud of?
3. What are your feelings and attitudes about death?
4. What has been the biggest disappointment in your life?
5. What is your most common sexual fantasy?
6. What have you done in your life that you feel most guilty about?
7. What characteristics of your best friend really bother you?

- That's the end of my questions. But is there anything else you'd like to add to those?

- Thanks for being willing to share your thoughts with me and support this study. To close out, please complete this short follow-up survey. I'll share the link in the chat box here. (https://cmu.ca1.qualtrics.com/jfe/form/SV_b41tamPmlQqNLhj?platform=video) Take as much time as you need. Your survey answers will not affect your compensation, and I won't have access to your responses.
- Assuming you finish that survey, you will receive your compensation of $10 as a bonus on Prolific within 48 hours. If you have any questions, you can always reach our team at interview-study@andrew.cmu.edu. I'll leave this chat window open a while longer in case you run into any problems.

Close: Update your spreadsheet and complete the follow-up survey:
https://cmu.ca1.qualtrics.com/jfe/form/SV_9EwD9bPaTZypkRD

Audio

Tips: Ensure that your environment does not have noise or distractions in the background. Because audio can require troubleshooting, set up early and test your setup. Be aware of the tone that is set by things like your tone of voice.

Set Up: Start the zoom meeting by clicking the link in your calendar event. Join with computer audio. Enable audio and video for both participants.

Interview Script

- Hi! Thanks for joining me for this interview. I'm [name]. As a reminder, this interview is being recorded.
- So you agreed to a consent form earlier. I just wanted to check whether you have any questions about that before we begin?
- Great! I'm going to ask you around 10 questions over the next 30 or so minutes. There's no wrong answers and everything you say will be kept confidential, so just try to answer honestly. Try to avoid sharing identifying information about yourself or others; I'm on a need-to-know basis. For example, feel free to use fake names or just an initial when telling stories about others.
- If there's ever a question you're not comfortable with or don't wish to answer, feel free to say so and we can just move on instead. Sound good?

1. And just as a warning, It might seem like the questions jump around, so don't be taken off guard. :) What are your favorite things to do in your free time?
2. What characteristics of yourself are you most proud of?
3. What are your feelings and attitudes about death?
4. What has been the biggest disappointment in your life?

5. What is your most common sexual fantasy?
6. What have you done in your life that you feel most guilty about?
7. What characteristics of your best friend really bother you?

- That's the end of my questions. But is there anything else you'd like to add to those?
- Thanks for being willing to share your thoughts with me and support this study. To close out, please complete this short follow-up survey. I'll share the link in the chat box here. (https://cmu.ca1.qualtrics.com/jfe/form/SV_b41tamPmlQqNLhj?platform=audio) Take as much time as you need. Your survey answers will not affect your compensation, and I won't have access to your responses.
- Assuming you finish that survey, you will receive your compensation of $10 as a bonus on Prolific within 48 hours. If you have any questions, you can always reach our team at interview-study@andrew.cmu.edu. I'll leave this chat window open a while longer in case you run into any problems.

Close: Update your spreadsheet and complete the follow-up survey: https://cmu.ca1.qualtrics.com/jfe/form/SV_9EwD9bPaTZypkRD

# Email

Tips: Be aware that it can be tricky to establish your tone of voice through this text-based medium; you may want to compensate with clarity. Mirror your interviewee's messaging style :). Part of the joy of email is that the asynchronicity allows extra time for the interviewee to reflect. Try to keep mental track of how much you're cumulatively spending time reading and writing.

Instructions: *Use the email that the participant provided in Calendly.* Add that email to your spreadsheet. Questions are sent in chunks. See the appendix for an example. The first rounds of logistical info will be sent at the scheduled interview start time. After you send the initial block of personal questions, you can allow responses to happen at leisure. However, you're expected to respond within 12 hours. If they don't respond to you within a day, send two additional emails every 24 hours to give them a nudge. After a week or so, you can mark them as a no-show. Reach out to us to let us know how far you go into the interview so we can decide how to allot compensation.

Most interviewees will be using an anonymous email address provided by the recruitment platform, Prolific. Unfortunately, this email address doesn't thread emails very nicely. Take care to send each question round to the correct email address, rather than Prolific's no-reply email address. When sending follow-up questions, make sure you're clear about which question your follow-up refers to.

Script: Each message block is marked with // to separate

- Hi! Thanks for joining me for this interview. I'm [name]. You agreed to a consent form earlier. I just wanted to check whether you have any questions about that before we

begin? And could you please give me your Prolific ID again? (We need to make sure we're getting payment to the correct person!) //

- Great! I'm going to ask you around 10 questions. I might send several questions at a time. I will reply to all your emails within 12 hours, and will usually reply faster than that. You're welcome to spend as much time as you like thinking, but I hope you'll reply in less than a day to my questions. If you don't, I'll send a quick reminder follow-up. Does that sound good to you?

  If there's ever a question you're not comfortable with or don't wish to answer, feel free to say so and we can just move on instead. Sound good? //

1. And just as a warning, It might seem like the questions jump around, so don't be taken off guard. :) What are your favorite things to do in your free time?
2. What characteristics of yourself are you most proud of?
3. What are your feelings and attitudes about death?
4. What has been the biggest disappointment in your life?

//

5. What is your most common sexual fantasy?
6. What have you done in your life that you feel most guilty about?
7. What characteristics of your best friend really bother you?

//

- That's the end of my questions. But is there anything else you'd like to add to those?

  Thanks for being willing to share your thoughts with me and support this study. To close out, please complete this short follow-up survey. I'll share the link in the chat box here. (https://cmu.ca1.qualtrics.com/jfe/form/SV_b41tamPmlQqNLhj?platform=email) Take as much time as you need. Your survey answers will not affect your compensation, and I won't have access to your responses.

  Assuming you finish that survey, you will receive your compensation of $10 as a bonus on Prolific within 48 hours. If you have any questions, you can always reach our team at interview-study@andrew.cmu.edu. Lastly, if you have privacy concerns about this interview, we recommend that you delete emails from your email provider's servers.

//

Close: Forward your chain to interview-study@andrew.cmu.edu. Make sure the Prolific ID of the participant made it into the chain. Update your spreadsheet and complete the follow-up survey: https://cmu.ca1.qualtrics.com/jfe/form/SV_9EwD9bPaTZypkRD

Chat

Tips: Feel free to copy-paste. Be aware that it can be tricky to establish your tone of voice through this text-based medium; you may want to compensate with clarity. Mirror your interviewee's messaging style :). To help maintain engagement, shorter, more frequent messages are better than longer, infrequent messages.

Set Up: Start the zoom meeting by clicking the link in your calendar event. Hover over the bottom toolbar & click the chat icon.

Interview Script

- Hi [name]! Thanks for joining me for this interview. I'm [name]. As a reminder, this interview is being recorded.
- So you agreed to a consent form earlier. I just wanted to check whether you have any questions about that before we begin?
- Great! I'm going to ask you around 10 questions over the next 30 or so minutes. There's no wrong answers and everything you say will be kept confidential, so just try to answer honestly. Try to avoid sharing identifying information about yourself or others; I'm on a need-to-know basis. :) For example, feel free to use fake names or just an initial when telling stories about others.
- If there's ever a question you're not comfortable with or don't wish to answer, feel free to say so and we can just move on instead. Sound good?
- If you need to pause to think, please just give us an indication so I'm not confused why you're not responding. If I accidentally cut you off with a new question, feel free to continue responding to the one you were thinking about.

8. And just as a warning, It might seem like the questions jump around, so don't be taken off guard. :) What are your favorite things to do in your free time, name?
9. What characteristics of yourself are you most proud of?
10. What are your feelings and attitudes about death?
11. What has been the biggest disappointment in your life?
12. What is your most common sexual fantasy?
13. What have you done in your life that you feel most guilty about?
14. What characteristics of your best friend really bother you?

- That's the end of my questions. But is there anything else you'd like to add to those?
- Thanks for being willing to share your thoughts with me and support this study. To close out, please complete this short follow-up survey. I'll share the link in the chat box here. (https://cmu.ca1.qualtrics.com/jfe/form/SV_b41tamPmlQqNLhj?platform=im) Take as

much time as you need. Your survey answers will not affect your compensation, and I won't have access to your responses.
- Assuming you finish that survey, you will receive your compensation of $10 as a bonus on Prolific within 48 hours. If you have any questions, you can always reach our team at interview-study@andrew.cmu.edu. I'll leave this chat window open a while longer in case you run into any problems. Thanks, name!

Close: Update your spreadsheet and complete the follow-up survey: https://cmu.ca1.qualtrics.com/jfe/form/SV_9EwD9bPaTZypkRD

Anonymous Chat

Tips: Feel free to copy-paste. Be aware that it can be tricky to establish your tone of voice through this text-based medium; you may want to compensate with clarity. Mirror your interviewee's messaging style :). To help maintain engagement, shorter, more frequent messages are better than longer, infrequent messages.

Set Up: Start the zoom meeting by clicking the link in your calendar event. Hover over the bottom toolbar & click the chat icon.

Interview Script

- Hi! Thanks for joining me for this interview. I'm [name]. As a reminder, this interview is being recorded.
- So you agreed to a consent form earlier. I just wanted to check whether you have any questions about that before we begin?
- Great! I'm going to ask you around 10 questions over the next 30 or so minutes. There's no wrong answers and everything you say will be kept confidential, so just try to answer honestly. Try to avoid sharing identifying information about yourself or others; I'm on a need-to-know basis. :) For example, feel free to use fake names or just an initial when telling stories about others.
- If there's ever a question you're not comfortable with or don't wish to answer, feel free to say so and we can just move on instead. Sound good?
- If you need to pause to think, please just give us an indication so I'm not confused why you're not responding. If I accidentally cut you off with a new question, feel free to continue responding to the one you were thinking about.

15. And just as a warning, It might seem like the questions jump around, so don't be taken off guard. :) What are your favorite things to do in your free time?
16. What characteristics of yourself are you most proud of?
17. What are your feelings and attitudes about death?
18. What has been the biggest disappointment in your life?
19. What is your most common sexual fantasy?
20. What have you done in your life that you feel most guilty about?

21. What characteristics of your best friend really bother you?

- That's the end of my questions. But is there anything else you'd like to add to those?
- Thanks for being willing to share your thoughts with me and support this study. To close out, please complete this short follow-up survey. I'll share the link in the chat box here. (https://cmu.ca1.qualtrics.com/jfe/form/SV_b41tamPmlQqNLhj?platform=im) Take as much time as you need. Your survey answers will not affect your compensation, and I won't have access to your responses.
- Assuming you finish that survey, you will receive your compensation of $10 as a bonus on Prolific within 48 hours. If you have any questions, you can always reach our team at interview-study@andrew.cmu.edu. I'll leave this chat window open a while longer in case you run into any problems. Thanks!

Close: Update your spreadsheet and complete the follow-up survey: https://cmu.ca1.qualtrics.com/jfe/form/SV_9EwD9bPaTZypkRD

Scheduled Survey

Set Up: This condition doesn't require any setup beyond the invitation. Around the scheduled start time, you should monitor your email to be able to respond to any questions. The interviewee will complete a survey version of the interview, so you're not needed to conduct it.

Close: Update your spreadsheet and complete the follow-up survey. Feel free to ignore any questions that aren't relevant. https://cmu.ca1.qualtrics.com/jfe/form/SV_9EwD9bPaTZypkRD

Survey

Set Up: This condition doesn't require any setup beyond the invitation. The interviewee will complete a survey version of the interview, so you're not needed to conduct it.

Close: Update your spreadsheet and complete the follow-up survey. Feel free to ignore any questions that aren't relevant. https://cmu.ca1.qualtrics.com/jfe/form/SV_9EwD9bPaTZypkRD

What to do if…

Interviewee is late/no-show

Check your email to see if they have sent you a message with an explanation. After 5 minutes, send the interviewee-late email template to remind them. After 15 minutes of waiting, fill out the interview follow-up questionnaire as best as you can and move on. In your spreadsheet, set them as "no-show"

If they don't reply in 24 hours, send them the interviewee-no-show email. If they still don't reply, after around 3 days, we'll consider them removed from the study. In your spreadsheet, set them as "removed no-show."

We have a three strikes rule; if they don't show the third time, feel free to give up on them.

I am late/no-show

Mistakes happen. If you're late or miss an interview, please don't try to hide that fact. If you're less than 15 minutes late, apologize and ask the interviewee if they're willing to continue or would prefer to reschedule. Make a note of lateness in your follow-up questionnaire.

If you miss an interview, send the interviewer-no-show email template as soon as possible.

Need to reschedule/cancel

The link to reschedule or cancel through Calendly can be found in your confirmation email or by logging into Calendly and looking at your Dashboard. If you're the one rescheduling, please make sure to send your interviewee an apology.

Audio or video isn't working

Use email or the Zoom chat feature to communicate with the participant. First, determine what client (web or desktop) and browser the interviewee is using. If they are using Internet Explorer, encourage them to use Chrome or Safari, or download the desktop client. Here's some common problems:

- **Hasn't joined audio**. This may be the case if they have no microphone icon by their name in the participants list. In the bottom left of their screen, they should click the "join audio" button and use the "Computer Audio" tab if possible.
- **Participant is muted or camera is disabled.** You can see this in the participant list if they have a small "X" over their camera or audio icon. To unmute or enable camera, they should hover over the bottom left of their screen and click the relevant mic or camera icon. You can also unmute participants by hovering over their name in the participants list.
- **Browser camera or microphone blocked.** This may be the case if they can see a red exclamation point above their camera or microphone icon in the bottom left. Check out instructions for Chrome, Safari, Firefox audio, or Edge audio.

- **Problem with device.** This is tougher to troubleshoot remotely. Ask them to try Googling their device, operating system and "test microphone/webcam." Ask them to try a different mic or camera, such as a USB camera or headphones with a mic.

If you've been troubleshooting for more than 10 minutes or the person is visibly frustrated, offer to reschedule the interview after they've resolved their connectivity issues. They can test their connectivity at any time at zoom.us/test. For compliance reasons, we are required to use Zoom for these interviews, so please don't try to use another platform as an alternative. Make sure to note your issues in the follow-up questionnaire and send us a note about the situation.

Other technical difficulties

In the case of difficulties like web connectivity, device failure, or server outage, your main strategy is to be as communicative as possible with the interviewee, reschedule the interview, and reach out to us to see if we can help. The link for rescheduling an interview can be found in your Calendly confirmation email or calendar event.

Interviewee was inappropriate/I feel unsafe

You are always welcome to disengage or exit the interview at any point. If an interview makes you feel unsafe or triggered, we strongly encourage you to disengage and reach out to us about the experience.

If the interviewee is being inappropriate but bearable you're welcome to try correcting their behavior. For example:

- "I know this interview involves intimate topics, but I'd appreciate it if you respect that this is a professional environment."
- "That language makes me uncomfortable. Let's move on to a different question."
- "I really appreciate your willingness to be open, but to protect both of us, I'd rather not hear about potentially illegal activity."

Interviewee has strong negative response

Because these interviews cover sensitive, personal topics, it's possible that an interviewee will have strong emotional responses to your questions. We've tried to write questions that elicit a reflective rather than traumatic experience, but digging up memories can still be deeply painful. We encourage you to keep a non-judgmental and compassionate stance to interviewee answers. Try to mirror the language that your interviewee uses to indicate respect for their position. For example, if they use language like "My girlfriend was really mean" to describe an experience that sounds like domestic abuse, keep using the words and phrases they prefer. If a question is visibly painful for them, remind them that they're welcome to skip any questions with no repercussions, or can take a break for a moment.

While interviews can sometimes be cathartic for interviewees, at the end of the day, you're not a trained therapist and are not responsible for their wellbeing. If someone asks for help, you are welcome to try and assist them to the best of your abilities and willingness, but ultimately you need to gently disengage. Thank them for their honesty and trust and move on. For example, "I'm sorry to hear that you're having negative thoughts. Thanks for being so open with me. Since I'm not really a therapist, I can't help you much. I wish I could. But I know there's lots of great resources if you Google the suicide hotline in your area." or "I'm sorry that question made you panic. I'm going to go ahead and end this call so you can take as much time as you need to care for yourself. Is there a trusted person you can call to lend you some support?"

Appendix

Configuring Calendly

- Log in to your Calendly account provided by the research team
- Configure Google Calendar integration
- Navigate to your account in the top right of the menu bar and select "Calendar Connection" from the drop-down.

[Image: screenshot of options dashboard highlighting calendar integrations]

Removed for licensing purposes.

Select the Google Calendar option:

[Image: screenshot of calendar integration options]

Removed for licensing purposes.

Login or select the assigned interviewer inbox account:

[Image: screenshot of initial sign-in step to integrate with the interviewer's calendar]

Removed for licensing purposes.

Allow integration access:

[Image: screenshot of options pertaining to sharing permissions for calendar integration]

Removed for licensing purposes.

# Email Templates

We recommend that you save these as a canned response in Gmail (instructions modified from NCSU).

1. Open Gmail
2. Click the Settings gear icon, then “Settings”>“Advanced” tab
3. Scroll to “Canned Responses” and enable
4. Save changes at bottom of page.
5. Return to your email and open a new message.
6. Copy-Paste a template from below
7. At the bottom of the window, click “... More Options” > “Canned Responses” > “Save Draft as Template” > “Save Draft as New Template”
8. Give the template the corresponding name (e.g., “Invitation to video interview for $10”). This will become the default subject line as well.

To use these templates in the future, open a new message in the compose window and select “More Options” > “Canned Responses.”

Video/Audio/Chat Invite

Subject: Invite to [video/audio/chat] interview for $10

Greetings,

You recently completed a screening survey for a 45-minute online interview and follow-up survey on sensitive topics with $10 compensation. I’m happy to say that you passed all our screening requirements, so I’d like to invite you to participate in an interview via [video/audio/instant message].

If you’d like to participate, please pick a slot that fits your schedule here.

Video:
https://calendly.com/interview-study[X]/interview-video?name=Do%20not%20provide%20your%20name&email=But%20please%20provide%20your%20Prolific%20email

Audio:
https://calendly.com/interview-study3/interview-audio?name=Do%20not%20provide%20your%20name&email=But%20please%20provide%20your%20Prolific%20email

Chat:

https://calendly.com/interview-study3/interview-chat

Anonymous Chat:

https://calendly.com/interview-study3/interview-anonymous-chat?name=Do%20not%20provide%20your%20name&email=But%20please%20provide%20your%20Prolific%20email

If none of those times work for you, feel free to reply to this email and we can try to find something that works for both of us. My name is [NAME], and I'll be your interviewer. If you have any questions or concerns, don't hesitate to reach out.

As a reminder, here's a summary of some information about the interview. I've also attached a copy of the consent form you already agreed to.

- **Sensitive topics**: This interview covers sensitive topics such as your thoughts & experiences with sex, death, and guilt. You can choose to skip any question in good faith without affecting your compensation.
- **Risks & Benefits**: The topics covered in this interview risk making you feel uncomfortable. There is also always a risk of a breach of research confidentiality. There may be no personal benefit from your participation in the study but the knowledge received may be of value to humanity.
- **Confidentiality**: We take measures to protect your confidentiality. Your answers in interviews and surveys will not be associated with any personal information you provide. Your full answers will only be shared with outside parties for the purposes of completing research, and if we share small portions of your answers, we will review them to make sure they do not personally identify you.
- **This research is voluntary**. You can refuse a question, stop the interview or withdraw from this study at any time, for any reason.

Thanks,

[NAME]

[ATTACH CONSENT FORM]

Email Invite

Subject: Invitation to participate in interview for $10

"Greetings,

You recently completed a screening survey for a 45-minute (cumulative) online interview and follow-up survey on sensitive topics with $10 compensation. I'm happy to say that you passed all our screening requirements, so I'd like to invite you to participate in an interview via email.

If you'd like to participate, please pick a slot that fits your schedule here:

https://calendly.com/interview-study3/interview-email?name=Do%20not%20provide%20your%20name&email=But%20please%20provide%20your%20Prolific%20email

We'll kick off the interview at the scheduled time, but once we get into the actual questions, you're welcome to reply at your leisure to the questions. If none of those times work for you, feel free to reply to this email and we can try to find something that works for both of us. My name is [NAME], and I'll be your interviewer. If you have any questions or concerns, don't hesitate to reach out.

As a reminder, here's a summary of some information about the interview. I've also attached a copy of the consent form you already agreed to.

- **Sensitive topics**: This interview covers sensitive topics such as your thoughts & experiences with sex, death, and guilt. You can choose to skip any question in good faith without affecting your compensation.
- **Risks & Benefits**: The topics covered in this interview risk making you feel uncomfortable. There is also always a risk of a breach of research confidentiality. There may be no personal benefit from your participation in the study but the knowledge received may be of value to humanity.
- **Confidentiality**: We take measures to protect your confidentiality. Your answers in interviews and surveys will not be associated with any personal information you provide. Your full answers will only be shared with outside parties for the purposes of completing research, and if we share small portions of your answers, we will review them to make sure they do not personally identify you.
- **This research is voluntary**. You can refuse a question, stop the interview or withdraw from this study at any time, for any reason.

Thanks,

[NAME]

[ATTACH CONSENT FORM]

Scheduled Survey Invite

Subject: Invitation to participate in interview for $10

"Greetings,

You recently completed a screening survey for a 45-minute online interview and follow-up survey on sensitive topics with $10 compensation. I'm happy to say that you passed all our screening requirements, so I'd like to invite you to participate in an interview via an open-ended survey interview.

If you'd like to participate, please pick a slot that fits your schedule here:

https://calendly.com/interview-study[X]/interview-survey?name=Do%20not%20provide%20your%20name&email=But%20please%20provide%20your%20Prolific%20email

You'll need to set aside time to complete the survey during that scheduled block. If none of those times work for you, feel free to reply to this email and we can try to figure something out that works. My name is [NAME], and I'll be your interviewer. If you have any questions or concerns, don't hesitate to reach out.

As a reminder, here's a summary of some information about the interview. I've also attached a copy of the consent form you already agreed to.

- **Sensitive topics**: This interview covers sensitive topics such as your thoughts & experiences with sex, death, and guilt. You can choose to skip any question in good faith without affecting your compensation.
- **Risks & Benefits**: The topics covered in this interview risk making you feel uncomfortable. There is also always a risk of a breach of research confidentiality. There may be no personal benefit from your participation in the study but the knowledge received may be of value to humanity.
- **Confidentiality**: We take measures to protect your confidentiality. Your answers in interviews and surveys will not be associated with any personal information you provide. Your full answers will only be shared with outside parties for the purposes of completing research, and if we share small portions of your answers, we will review them to make sure they do not personally identify you.
- **This research is voluntary**. You can refuse a question, stop the interview or withdraw from this study at any time, for any reason.

Thanks,

[NAME]

[ATTACH CONSENT FORM]

Survey Invite

Subject: Invitation to participate in interview for $10

“Greetings,

You recently completed a screening survey for a 45-minute online interview and follow-up survey on sensitive topics with $10 compensation. I'm happy to say that you passed all our screening requirements, so I'd like to invite you to participate in an interview via an open-ended survey interview.

If you'd like to participate, please complete this within the next two weeks:

https://cmu.ca1.qualtrics.com/jfe/form/SV_3mT8tHp2fk0hiEB

My name is [NAME], and I'll be your interviewer. If you have any questions or concerns, don't hesitate to reach out.

As a reminder, here's a summary of some information about the interview. I've also attached a copy of the consent form you already agreed to.

- **Sensitive topics**: This interview covers sensitive topics such as your thoughts & experiences with sex, death, and guilt. You can choose to skip any question in good faith without affecting your compensation.
- **Risks & Benefits**: The topics covered in this interview risk making you feel uncomfortable. There is also always a risk of a breach of research confidentiality. There may be no personal benefit from your participation in the study but the knowledge received may be of value to humanity.
- **Confidentiality**: We take measures to protect your confidentiality. Your answers in interviews and surveys will not be associated with any personal information you provide. Your full answers will only be shared with outside parties for the purposes of completing research, and if we share small portions of your answers, we will review them to make sure they do not personally identify you.
- **This research is voluntary**. You can refuse a question, stop the interview or withdraw from this study at any time, for any reason.

Thanks,

[NAME]

[ATTACH CONSENT FORM]

Interviewee-late

Send 5 minutes after interview start time.

Subject: Is now still a good time for your interview?

"Greetings,

I'm waiting to conduct the interview we scheduled for [X/X at X:00]. Please check your confirmation email for instructions on accessing the interview platform. If you are having issues, feel free to reach out. If you need to reschedule, you can send me a note and pick a new time slot using the reschedule link provided in your confirmation email. I'll be waiting for around 10 more minutes.

Thanks,

[name]"

Interviewee-no-show

"Greetings,

I noticed you missed the interview we had scheduled for [X/X at X:00]. If you need to reschedule, you can send me a note and pick a new time slot using the reschedule button provided in your confirmation email. If you changed your mind and want to cancel, you can do so here in the link provided in your confirmation email. If I don't hear from you in the next 3 days, I'll remove you from our participation list.

Thanks,

[name]"

Interviewer-no-show

You don't have to follow this template exactly. Feel free to adjust it to better fit your voice and circumstances.

Subject: Apologies for missing our interview

Greetings,

I just realized that I didn't show up to the interview we scheduled [yesterday]. [Was there a reason?] I apologize for disrespecting your time. If you're willing to try again, you can pick another time slot at [link from Calendly email or event]. [I've taken some steps to ensure I won't miss future interviews.] Again, I'm so sorry for this frustrating mistake. Please let me know if you have any concerns.

Thanks,

Example Email Interview

Read this in reverse. Participant responses in italics.

Sample interview from pilot study.

Removed for confidentiality.

Copy of Your Consent Form

You can find a copy in your folder.
