# Supplementary material for: Audio, video, chat, email, or survey: How much does online interview mode matter?
Source: PLoS One. 2022 Feb 22;17(2):e0263876. doi: 10.1371/journal.pone.0263876 (PMC8863281; doi:10.1371/journal.pone.0263876)
Supplement: S2 Protocol — Handbook containing qualitative coding instructions and procedures. (PDF) [file pone.0263876.s002.pdf]

*We'll be writing this and updating it as we go, deciding norms and details.*

# Contents


[Contents](#)

[Project Overview](#)

[Analysis](#)
- [Overview of Exploratory Content Analysis](#)
- [Coding Reminders](#)
- [Research Questions](#)
  - [Primary RQs](#)
  - [Sub RQs](#)
- [Deliberation meetings](#)
- [Final Round - Consensus Coding](#)
- [Spreadsheet Wrangling](#)
- [Follow-up Question Numbering](#)
- [Readings](#)
- [Lab Notebooks](#)
- [Terminology](#)

[Logistics](#)
- [Contact Info](#)
- [Hours](#)
- [Expectations of RAs](#)
- [Expectations you can have of us](#)
- [Take Care of Yourself](#)
- [Technology](#)
- [Human Subjects Research Certification](#)
- [Accessing Academic Materials](#)

[Extra References](#)
- [Interview script](#)
- [How to make a clean coding sheet](#)

# Project Overview

Interviews are a great way to generate rich data about people's nuanced lives. As a qualitative method, interviewing can capture a moment in time for a person's perspective on their stories, experiences, memories, and attitude. However, interviews are not necessarily the best method for collecting data across *time*. They usually ask interviewees to remember stories or experiences on-the-spot, so they can be affected by recency bias or retell an experience in a different way depending on spurious factors like their current emotional state. If I ask you to tell me about your childhood in an interview, your answers may vary drastically depending on whether you got in a fight with your mother yesterday. In addition, there's no research available for understanding how doing *online* interviews might expect the experience of interviewing.

To better understand how people reflect on and reason about their experiences, we conducted around 150 interviews with people focused on sensitive topics like sex, death, friendship, and guilt. The questions were picked to spark personal reflection on sensitive topics. (See the script below.)

Some example relevant exploratory research questions:

- If asked to recall a moment in time, from which eras of life do people tend to recall memories?
- When considering personal development, how much change in themselves do people perceive?
- How much do people practice self-distancing during interviews?

However, for this stage of analysis, we're interested in understanding these reflections as though we were researchers interested in each interview question on its own. For example, for the question "What are your feelings and attitudes about death?" our research question to focus on will be "What are our participants' general feelings and attitudes about death?"

# Analysis

## 5-35Overview of Exploratory Content Analysis

Our content analysis will focus on understanding, summarizing, and finding patterns in the 'first layer' *content* of the text. There are many other layers and dimensions that could be analyzed (for example, think about how you might analyze a poem for a class; looking at it's form, word choice, metaphor usage, meta-meaning), but in this stage we will try to focus on the content without 'reading into' the transcripts too much.

You'll be working in pairs, analyzing one question at a time. Collaboration makes for richer qualitative analysis. You certainly don't always have to agree, but please *do* document your disagreements.

Here's a rough outline of the process

*Codebook drafting*

1. Pick on question. Take a sample of the responses (~250 rows)
2. On your own
   a. skim the responses
   b. go back & for each response highlight and summarize content *related to the research Qs*
   c. if there's obvious ways to consolidate your codes (e.g., "fear" and "scared" can be combined), do so! you don't need to go too deep though
3. With your partner
   a. compare the initial codes you produced
   b. talk through and identify patterns, themes, and relationships
   c. standardize language (e.g., "bullied" vs "bullying")
   d. Make the first codebook draft; Aim to end up with 5-35 codes grouped under several categories

*Applying codes*

4. On your own
   a. take a new set of responses (~150 rows), and apply your codebook to them
   b. make note of possible holes in the codebook, points of ambiguity, places where you weren't sure what codes applied; interesting observations
5. With your partner (*Deliberation meetings*)
   a. Compare codes ; discuss discrepancies
   b. Disagreements suggest that there's (1) ambiguous content; (2) an ambiguous codebook; or (3) just subjective differences in how you two interpret the text
   c. Adjust the codebook as needed
6. (Repeat steps 4 & 5 as many times as needed)
   a. "As needed" means: repeat until you & your partner agree ~75% of the time
   b. Or, when your disagreements mostly stem from your individual subjective reads of the text rather than ambiguity in the codebook
7. Code the entire response set with your final codebook (usually ~1300 rows)
8. With your partner, do the final round (*Consensus code*)
9. Brain dump in your lab notebook anything you learned. Here's some optional prompts
   a. What do you know about your Research Qs that you didn't before?
   b. What was the most surprising thing in your text? The most boring? The most predictable?
   c. If you were going to sketch out or draw your working answer to your research Qs, what would it look like?

    d. This was one exploratory round of coding. If you were going to do a second round with a new, more specific research Q, what would you focus on?
10. Congrats! You're ✨officially done✨

## Coding Reminders

- Code in your personal spreadsheet; try not to peek at your partners' codes until you meet to discuss
- You can use more than one code per row. Always separate them with a comma or semicolon. (Just be consistent.)
- Not every row needs a code.
- While you code, have your journal open in another window so you can document times when you're confused or unsure about what codes fit, or any trends you're noticing.
- If it doesn't seem like any codes fit, but the content is relevant to the research question, make a note to discuss with your partner.
- At the end, codes will be considered at the response level rather than the utterance level. As a result, you only need to mark each code at most once per response. Try to mark codes the first time they appear in the text. If codes are off by an utterance or two, it's not a big deal.

## Research Questions

Each Question has its own set of research questions to work with. The Primary RQ is related to the individual question.

### Primary RQs

| **Question** | **Primary RQ**<br>*And a sub-Q to keep in mind.* |
|---|---|
| Free Time | What are Americans' favorite things to do in their free time? |
| Pride | What characteristics are Americans most proud of in themselves?<br><br>*What makes something worth being proud of?*<br>*What makes up pride?* |
| Death | What are Americans' feelings and attitudes about death? |
| Disappointment | What are the biggest disappointments in Americans' lives?<br><br>*What makes up disappointment?* |

| | |
|---|---|
| Sexual fantasy | What sexual fantasies do Americans commonly have?<br><br>*How do people reply if they don't commonly have sexual fantasies? Or don't want to answer?* |
| Guilt | What have Americans done that they feel most guilty about?<br><br>*What makes an action guilty?* |
| Best Friend | What characteristics of best friends do Americans get bothered by?<br><br>*What are people bothered by in friendships?*<br>*How do people reply if they don't have a best friend?* |

## Sub RQs

For each question, we will also be considering the follow set of sub-RQs. These *may or may not apply* to your question and text. If you don't see them arise in the text while you're open-coding, then feel free to set them aside!

Time

- Is there a moment/event/time period/life era highlighted? Any durational notes? ("for three years" ; "always")
- Any moments of change?
- Is there a notable focus on the present or the past?

Distance (only focus on these if they seem obvious/relevant)

- Does the participant talk about their past self as though they were a different person?
- Does the participant say they avoid thinking about an event/time? (avoidance)
- Does the participant become emotional while recounting an event/time? (embarrassed, emotional, crying)
- Does the participant just recount events or do they learn/express possibly new things during that (reconstructing)?
- If there's a conflict/uncertainty, does it resolve?

# Deliberation meetings

Each round of draft coding and resolution can be thought of as an assessment of your *codebook*, rather than a test of you and your partner. The goal of resolution meetings is to discuss confusions, anomalies, struggles.

If you and your partner differ in your codes, one a few things could be happening:

- someone made a simple mistake
- the codebook is ambiguous
- the text is ambiguous / hard to parse
- you & your partner have equally valid interpretations of the text

Your first task is usually to figure out which bullet point applies. If it's one of the first two bullet points, then try to come to a resolution on what needs to change. If it's one of the second two bullet points, then see what you might learn from each other, but don't get too stuck!

# Final Round - Consensus Coding

Once you feel like your codebook is really solid, you and your partner will do step 7 & 8 from the overview, coding the whole data set individually. Then, you'll meet one last time to do consensus coding. Unlike previous deliberation meetings focused on interrogating the codebook, your focus is now on picking the official, final codes.

Do a final sweep checking mainly for differences. Did you find...

- A typo? Just make the correction
- A minor row difference? (e.g., I assigned 'kind' to row 30 & you assigned it later in the response, in row 42) Just pick the first occurrence.
- You & your partner have different interpretations of the text? That's fine. Spend <1 min arguing your position. Often, you'll just combine both codes as valid interpretations.
- A possible fault in your codebook? Use the *other* tag if it makes sense. Make note of the codebook hiccup in your lab journal. If this happens often, it's a sign that you're not ready to do a final round.

Final/combined codes go in a new column, separated by a comma or semicolon.

# Spreadsheet Wrangling

# Follow-up Question Numbering

At some point during your coding process, make sure you tag follow-up questions for each Interviewer utterance. This should go in a separate column in your spreadsheet.

| | |
|---|---|
| initial Question | 0 |
| follow-up #1 | 1 |
| follow-up #2 ... | 2 ... |
| technical difficulty | TECH |
| anything else random / unrelated to the RQs | OTHER |

Sometimes, Interviewers follow up with comments that aren't exactly questions. Number the utterances that seem like they prompt the interviewee in a new or deeper direction. If the interviewer is just talking about themselves or vaguely agreeing, count it as *OTHER*. If you're really stuck, just put a **?,** tag me to look at it, and move on!

You and your partner don't need to deliberate on these or anything. This task is intended to be quick!

# Readings

We're doing a modified content analysis for this stage of the study, so not everything in these readings will apply in our case!

Chapter 11 "Analyzing Qualitative Data." from *Research Methods in Human-Computer Interaction*. by Jonathan Lazar, Jinjuan Heidi Feng, Harry Hochheiser. Elsevier Science & Technology. 2017.
link to interactive e-book (needs library VPN) / link to chapter pdf

Erlingsson, Christen, and Petra Brysiewicz. "A hands-on guide to doing content analysis." *African Journal of Emergency Medicine* 7.3 (2017): 93-99.
link / link to pdf

## Lab Notebooks

Each member of the analysis team has their own lab notebook (an idea borrowed from the hard sciences, where chemists documented their labwork). This is a place to keep your notes as you work. Qualitative analysis is highly *process-driven,* so keeping notes about your process is very helpful in the long term. We won't be scrutinizing or monitoring these, but we might review/reference them when we're writing a Analysis Method section in a paper someday.

In my lab notebooks, I try to make an entry every time I think or do something for this project. Rather than it just being a work log (e.g., "I coded 20 questions today", though that can be helpful for logging your hours in Workday later), I try to write down my:

- questions
- decisions
- observations
- summary of conversations

Many times in research, my lab notes have saved my ass when I'm questioning myself and need to remember the justification for a decision I made weeks ago.

## Terminology

| | |
|---|---|
| Question | The actual question the interviewer asked, (e.g., 'what are your feelings about death?') |
| Research Question (RQ) | A high-level question we're interested in exploring from our data (e.g., 'What are Americans' general attitudes about death?') |
| Response | The interviewee's response to a question, including relevant follow-up Qs |
| Transcript | The text from one entire interview, with all Qs & responses |
| Utterance | One row of a response (usually a line or a paragraph) |
| Code | A qualitative code is a subjective unit that helps us organize what our data is 'all about' (e.g., fear, acceptance, resigned) |
| Category | A category is a set of codes that are related in a common theme (e.g., emotions-around-death) |
| Codebook | The set of codes that apply to a single question |

| | |
|---|---|
| Inter-rater reliability | A quantitative measurement of how much coders agree on how to interpret a text with a given codebook |

# Extra References

## Interview script

Hi! Thanks for joining me for this interview. I'm [name]. As a reminder, this interview is being recorded.

So you agreed to a consent form earlier. I just wanted to check whether you have any questions about that before we begin?

Great! I'm going to ask you around 10 questions over the next 30 or so minutes. There's no wrong answers and everything you say will be kept confidential, so just try to answer honestly. Try to avoid sharing identifying information about yourself or others; I'm on a need-to-know basis. For example, feel free to use fake names or just an initial when telling stories about others.

If there's ever a question you're not comfortable with or don't wish to answer, feel free to say so and we can just move on instead. Sound good?

1. And just as a warning, It might seem like the questions jump around, so don't be taken off guard. :) What are your favorite things to do in your free time?
2. What characteristics of yourself are you most proud of?
3. What are your feelings and attitudes about death?
4. What has been the biggest disappointment in your life?
5. What is your most common sexual fantasy?
6. What have you done in your life that you feel most guilty about?
7. What characteristics of your best friend really bother you?
8. That's the end of my questions. But is there anything else you'd like to add to those?
