# Supplementary material for: Audio, video, chat, email, or survey: How much does online interview mode matter?
Source: PLoS One. 2022 Feb 22;17(2):e0263876. doi: 10.1371/journal.pone.0263876 (PMC8863281; doi:10.1371/journal.pone.0263876)
Supplement: S3 Protocol — Transcript of the screening survey taken by potential participants to determine their eligibility. (PDF) [file pone.0263876.s003.pdf]

Interviewee Survey

Demographics

1) What is your age?

- ○ Under 18 (1)
- ○ 18 - 24 (2)
- ○ 25 - 34 (3)
- ○ 35 - 44 (4)
- ○ 45 - 54 (5)
- ○ 55 - 64 (6)
- ○ 65 - 74 (7)
- ○ 75 - 84 (8)
- ○ 85 or older (9)

---

2) Which gender do you identify most with?

- ○ Man (1)
- ○ Woman (2)
- ○ Non-binary or genderqueer (3)
- ○ Prefer to self-describe (5) ____
- ○ Prefer not to comment (4)

3) What best describes your current marital status?

- ○ Married (1)
- ○ Widowed (2)
- ○ Divorced (3)
- ○ Separated (4)
- ○ Never married (5)

4) What is your racial and/or ethnic identity?

____

____

____

____

____

5) What do you consider to be your cultural background? It is okay to have no answer or more than one answer.

____

____

____

____

____

6) Prolific ID

____

Logistics

7) Do you have reliable access to a computer to use for the interview? (Does not include mobile devices.)

- ○ Yes (1)
- ○ No (2)

8) Does your keyboard have a number pad?

- ○ Yes (1)
- ○ No (2)
- ○ I am not sure (3)

9) Do you have access to a webcam?

- ○ Yes (1)
- ○ No (2)

10) Do you have a Skype account?

- ○ Yes (1)
- ○ No (2)

11) What types of online interview are you willing to complete? Select all platforms that apply.

- ☐ online video call (2)
- ☐ online audio call (3)
- ☐ email (1)
- ☐ instant message (4)
- ☐ open-ended online survey (5)

12) Are you willing to conduct an interview where the video is recorded?

- ○ Yes (1)
- ○ No (2)

13) Will you have access to a private, quiet room for the interview?

- ○ Yes (1)
- ○ No (2)

14) What social media platforms have you used in the past month?

- ☐ Facebook (1)
- ☐ Instagram (2)
- ☐ Twitter (3)
- ☐ Pinterest (4)
- ☐ Reddit (5)
- ☐ Qzone (6)
- ☐ Weibo (7)
- ☐ Other (please specify) (8) ____

---

15) Where do you complete Prolific tasks most often?

- ○ Home (1)
- ○ Office space (4)
- ○ Coffee shop (2)
- ○ Library (5)
- ○ Public transportation (3)
- ○ Other - please specify: (6) ____

---

16) Please describe the environment where you typically complete Prolific tasks.

____

____

____

____

____

Typing Test

JS

17) This is a one minute typing test. It is for informational purposes and will not affect your eligibility. Please type the text under the text box into the text box. The test will begin when you start typing.

____

____

____

____

____

---

18) Typing Test Issue?

- ▢ The typing test isn't working for me. (1)
- ▢ I can't complete this for accessibility reasons. (4)

Eligibility - Successful Applicants

This block is shown to participants who are eligible for the interview study. To be eligible the participant:

1) Did not select “Under 18” (question 1)
2) Indicated they had reliable access to a computer (question 7)
3) Were willing to participate in a recorded video interview (question 12)
4) Selected they were willing to participate in all types of interviews (question 11)

We're happy to inform you that you may be eligible to participate in a research interview on sensitive topics. We will complete a 30 minute interview with a 15-minute follow-up survey, for a total of 45 minutes. You will receive $10 in compensation, which we will add as Prolific bonus on this survey task. If you choose not to participate, you will still receive compensation for this screening survey.

Here’s a summary of some information to help you make an informed decision about participating:

Sensitive topics: This interview covers sensitive topics such as your thoughts & experiences with sex, death, and guilt. You can choose to skip any question in good faith without affecting your compensation.

Risks & Benefits: The topics covered in this interview risk making you feel uncomfortable. There is also always a risk of a breach of research confidentiality. There may be no personal benefit from your participation in the study but the knowledge received may be of value to humanity.

Confidentiality: We take measures to protect your confidentiality. Your answers in interviews and surveys will not be associated with any personal information you provide. Your full answers will only be shared with outside parties for the purposes of completing research, and if we share small portions of your answers, we will review them to make sure they do not personally identify you.

This research is voluntary. You can refuse a question, stop the interview or withdraw from this study at any time, for any reason.

---

19) Are you interested in learning more about participating in this interview?

○ Yes. (1)

○ No. (You will still receive compensation for completing this survey.) (2)

Online Consent Form

This is only shown to participants who were eligible selected “Yes” to question 19 indicating willingness to participate in the study.

Interviewee Online Consent

This interview is part of a research study conducted by Maggie Oates at Carnegie Mellon University, under the supervision of Professor Lorrie Cranor. It is funded by the Carnegie Corporation of New York.

Summary
This study is an online interview followed by a short closing survey, and will last approximately 45 minutes. If you are selected for an interview, it will cover sensitive topics, such as your experiences with topics like sex, death, or guilt, which may make you feel uncomfortable. You’re welcome to skip any question in good faith without affecting your compensation. You can also choose to stop the interview at any time. We take measures to protect your identity and confidentiality. If you are selected for participation and complete both the interview and closing survey, you’ll be compensated $10. Agreeing to this consent form does not guarantee that you’ll be selected to complete an interview.

Purpose
The purpose of the research is to improve online interview methods on sensitive topics. This work could help improve future research important to public interest that focuses on difficult topics like grief or drug use.

Procedures
You will schedule an interview using an online calendar. To complete the 30-45 minute interview, we ask that you find a private space with reliable Internet access and without distractions. Within 24 hours of the interview, you will receive information about how to access the interview platform, which could be an interview over video chat, audio chat, instant messaging, email, or a survey interview. At the end of the interview, you will be asked to complete a short follow-up survey about your interview experience.

Interviewee Participant Requirements
Interviewee participation in this study is limited to English-speakers who are age 18 and older and reside in the US.

Risks
This interview will cover sensitive topics, such as your experiences with topics like sex, death, or guilt, which may make you feel uncomfortable. There is also a risk of breach of confidentiality, where third-parties could get access to your interview or interview records. To mitigate that risk, we do not associate your interview data with your personally identifiable data, and ensure that the transmission and storage of your information is done securely.

Benefits
There may be no personal benefit from your participation in the study but the knowledge received may be of value to humanity.

Compensation & Costs

If you complete both the interview and the follow-up survey, you will be compensated $10 as cash bonus through Prolific Academic. If you fail to complete either, there will be no partial compensation. There will be no cost to you if you participate in this study.

Data Collection

To mitigate the risks of a breach of privacy, please do not share any personally-identifiable information about yourself or any other person during the interview. For example, do not use another person's real name when answering interview questions.

Personally-identifiable data collection: We will collect and use your email address to communicate with you if you provide it. We will not associate your interview data with your email address.

Third-party data collection and storage: We have reviewed the privacy policies of the third-party platforms we use to schedule and conduct online interviews, and all the platforms may collect and store your IP address or information about you from preexisting cookies. Video, audio, and chat content may be stored in the cloud by third-parties for a short duration before being deleted. The security and privacy of email interviews depends in part on the practices of your email provider.

Audio transcription: For video and audio interviews, we may share all or part of your interview recordings with third-party services for the purpose of transcription. See the "Confidentiality" section for more information.

Confidentiality

By participating in this research, you understand and agree that Carnegie Mellon may be required to disclose your consent form, data and other personally identifiable information as required by law, regulation, subpoena or court order. Otherwise, your confidentiality will be maintained in the following manner:

Your data and consent form will be kept separate. Your consent form will be stored in a secure location on Carnegie Mellon property and will not be disclosed to third parties. By participating, you understand and agree that the data and information gathered during this study may be used by Carnegie Mellon and published and/or disclosed by Carnegie Mellon to others outside of Carnegie Mellon. However, your name, address, contact information and other direct personal identifiers will not be mentioned in any such publication or dissemination of the research data and/or results by Carnegie Mellon. Note that per regulation all research data must be kept for a minimum of 3 years.

For video and audio interviews, we may also share all or part of your interview recordings with third-party transcription services, who need to store portions of your interview. Transcription partners may use segments of audio to improve their services automatically. After transcription is complete, we will delete your recording and transcript from these services. Wherever possible, we will remove identifiable information from recordings before transcription.

Right to Ask Questions & Contact Information

If you have any questions about this study, you should feel free to ask them by contacting the Principal Investigator now at Maggie Oates, a PhD Student in the Institute for Software Research at 5000 Forbes Ave, Pittsburgh PA 15213. You can reach her at moates@cmu.edu, 412-268-7534, or the entire study team at interview-study@andrew.cmu.edu. If you have questions later, desire additional information, or wish to withdraw your participation please contact the Principal Investigator by mail, phone or e-mail in accordance with the contact information listed above.

If you have questions pertaining to your rights as a research participant; or to report concerns to this study, you should contact the Office of Research Integrity and Compliance at Carnegie

Mellon University. Email: irb-review@andrew.cmu.edu . Phone: 412-268-1901 or 412-268-5460.

Voluntary Participation
Your participation in this research is voluntary. You may discontinue participation at any time during the research activity. You may print a copy of this consent form for your records. Download a copy of this online consent form.

---

20) I am age 18 or older.

○ Yes (1)

○ No (2)

---

21) I have read and understand the information above.

○ Yes (1)

○ No (2)

---

22) I want to participate in this research as an interviewee participant, and want to continue with the possible interview and closing survey.

○ Yes (1)

○ No (2)

○ I need more information to decide. (4)

Consent Denied

This is shown to participants who indicated either:

1) They were under 18 (question 20)
2) They did not read and understand the consent form (question 21)

3) They did not want to participate (question 22)

You have indicated that you do not wish to further participate in this study. Thanks for taking this survey.

Hit "next" to complete your study and return to Prolific.

Consent Wants Additional Information

This is shown to participants if they responded “I need more information to decide” to question 22.

23) Thanks for requesting more information. We want to help you make an informed decision. Would you like us to reach out to you via Prolific with more information?

○ Yes (1)

○ No (2)

24) Do you have a specific question or point of confusion?

____

____

____

____

____

Thanks for taking this survey. Hit "next" to complete your study and return to Prolific.

Consent Completed

Thank you for your interest and for completing the consent process. If you are selected for participation, we will send you a message in the next few weeks via Prolific.

Hit "next" to complete your study and return to Prolific.

Ineligible

This is presented to participants who were deemed ineligible for the study. These participants were not shown the previous blocks related to willingness to participate and the consent form. Participants were determined to be ineligible if they either:

1) Selected “Under 18” (question 1)
2) Indicated they did not have reliable access to a computer (question 7)
3) Were not willing to participate in a recorded video interview (question 12)
4) Did not select willingness to participate in all interview options (question 11)

Thanks for your interest. If you are selected for participation, we will send you a message in the next few months via Prolific.

Hit "next" to complete your study and return to Prolific.
