# Supplementary material for: Audio, video, chat, email, or survey: How much does online interview mode matter?
Source: PLoS One. 2022 Feb 22;17(2):e0263876. doi: 10.1371/journal.pone.0263876 (PMC8863281; doi:10.1371/journal.pone.0263876)
Supplement: S4 Protocol — Transcript of the post-interview survey taken by the interviewee after their interview. (PDF) [file pone.0263876.s004.pdf]

Interviewee Post-Interview Survey

Interview Platform

1) What kind of interview did you participate in?

- ○ video (1)
- ○ audio (2)
- ○ instant message (3)
- ○ email (4)
- ○ survey (5)

Social Desirability

For the following questions, please indicate how much you agree or disagree with the statement.

2) I have not always been honest with myself.

- Strongly agree (1)
- Agree (2)
- Somewhat agree (3)
- Neither agree nor disagree (4)
- Somewhat disagree (5)
- Disagree (6)
- Strongly disagree (7)

---

3) I always know why I like things.

- Strongly agree (1)
- Agree (2)
- Somewhat agree (3)
- Neither agree nor disagree (4)
- Somewhat disagree (5)
- Disagree (6)
- Strongly disagree (7)

---

4) It's hard for me to shut off a disturbing thought.

- Strongly agree (1)
- Agree (2)
- Somewhat agree (3)
- Neither agree nor disagree (4)
- Somewhat disagree (5)
- Disagree (6)
- Strongly disagree (7)

---

5) I never regret my decisions.

- Strongly agree (1)
- Agree (2)
- Somewhat agree (3)
- Neither agree nor disagree (4)
- Somewhat disagree (5)
- Disagree (6)
- Strongly disagree (7)

---

6) I sometimes lose out on things because i can't make up my mind soon enough.

- Strongly agree (1)
- Agree (2)
- Somewhat agree (3)
- Neither agree nor disagree (4)
- Somewhat disagree (5)
- Disagree (6)
- Strongly disagree (7)

---

7) I am a completely rational person.

- Strongly agree (1)
- Agree (2)
- Somewhat agree (3)
- Neither agree nor disagree (4)
- Somewhat disagree (5)
- Disagree (6)
- Strongly disagree (7)

---

8) I am very confident of my judgments.

- Strongly agree (1)
- Agree (2)
- Somewhat agree (3)
- Neither agree nor disagree (4)
- Somewhat disagree (5)
- Disagree (6)
- Strongly disagree (7)

---

9) I have sometimes doubted my ability as a lover.

- Strongly agree (1)
- Agree (2)
- Somewhat agree (3)
- Neither agree nor disagree (4)
- Somewhat disagree (5)
- Disagree (6)
- Strongly disagree (7)

---

10) I sometimes tell lies if I have to.

- Strongly agree (1)
- Agree (2)
- Somewhat agree (3)
- Neither agree nor disagree (4)
- Somewhat disagree (5)
- Disagree (6)
- Strongly disagree (7)

---

11) I never cover up my mistakes.

- Strongly agree (1)
- Agree (2)
- Somewhat agree (3)
- Neither agree nor disagree (4)
- Somewhat disagree (5)
- Disagree (6)
- Strongly disagree (7)

---

12) There are occasions when I have taken advantage of someone.

- Strongly agree (1)
- Agree (2)
- Somewhat agree (3)
- Neither agree nor disagree (4)
- Somewhat disagree (5)
- Disagree (6)
- Strongly disagree (7)

---

13) I sometimes try to get even rather than forgive and forget.

- Strongly agree (1)
- Agree (2)
- Somewhat agree (3)
- Neither agree nor disagree (4)
- Somewhat disagree (5)
- Disagree (6)
- Strongly disagree (7)

---

14) I have said something bad about a friend behind their back.

- Strongly agree (1)
- Agree (2)
- Somewhat agree (3)
- Neither agree nor disagree (4)
- Somewhat disagree (5)
- Disagree (6)
- Strongly disagree (7)

---

15) When I hear people talking privately, I avoid listening.

- Strongly agree (1)
- Agree (2)
- Somewhat agree (3)
- Neither agree nor disagree (4)
- Somewhat disagree (5)
- Disagree (6)
- Strongly disagree (7)

---

16) I never take things that don't belong to me.

- ◯ Strongly agree (1)
- ◯ Agree (2)
- ◯ Somewhat agree (3)
- ◯ Neither agree nor disagree (4)
- ◯ Somewhat disagree (5)
- ◯ Disagree (6)
- ◯ Strongly disagree (7)

---

17) I don't gossip about other people's business.

- ◯ Strongly agree (1)
- ◯ Agree (2)
- ◯ Somewhat agree (3)
- ◯ Neither agree nor disagree (4)
- ◯ Somewhat disagree (5)
- ◯ Disagree (6)
- ◯ Strongly disagree (7)

Rapport & Trust (Non-Survey Conditions)

This section was shown only to participants who indicated they complete a non-survey (audio, video, email, chat) interview in question 1. A similar section, with slightly different wording, was presented to participants in the survey conditions.

18) How often did the interviewer seem ill at ease?

- During all or most of the interview (1)
- At a number of points during the interview (2)
- At one or two points during the interview (3)
- Never (4)

19) How often did you feel ill at ease during the interview?

- During all or most of the interview (1)
- At a number of points during the interview (2)
- At one or two points during the interview (3)
- Never (4)

20) How motivated did you feel you were during the interview?

- Very motivated (1)
- Motivated (2)
- Neither particularly motivated nor unmotivated (3)
- Unmotivated (4)
- Very unmotivated (5)

21) How attentive was the interviewer during the interview?

- Very attentive (1)
- Attentive (2)
- Neither particularly attentive nor detached (3)
- Detached (4)
- Very detached (5)

---

22) How sensitive was the interviewer to your responses and concerns?

- Very sensitive (1)
- Sensitive (2)
- Neither particularly sensitive nor insensitive (3)
- Insensitive (4)
- Very insensitive (5)

---

23) How supportive was the interviewer in giving feedback during the interview?

- Very supportive (1)
- Supportive (2)
- Neither particularly supportive nor unsupportive (3)
- Unsupportive (4)
- Very unsupportive (5)

---

24) How encouraged did you feel to make additional comments?

- Very encouraged (1)
- Encouraged (2)
- Neither particularly encouraged nor discouraged (3)
- Discouraged (4)
- Very discouraged (5)

Rapport & Trust (survey)

This section was shown only to participants who indicated they complete a survey (scheduled or non-scheduled) interview in question 1. A similar section, with slightly different wording, was presented to participants in the non-survey conditions.

25) How often did you feel ill at ease during the survey interview?

- During all or most of the interview (1)
- At a number of points during the interview (2)
- At one or two points during the interview (3)
- Never (4)

26) How motivated did you feel you were during the survey interview?

- Very motivated (1)
- Motivated (2)
- Neither particularly motivated nor unmotivated (3)
- Unmotivated (4)
- Very unmotivated (5)

---

27) How sensitive was the survey to your responses and concerns?

- Very sensitive (1)
- Sensitive (2)
- Neither particularly sensitive nor insensitive (3)
- Insensitive (4)
- Very insensitive (5)

---

28) How supportive was the survey in giving feedback during the interview?

- Very supportive (1)
- Supportive (2)
- Neither particularly supportive nor unsupportive (3)
- Unsupportive (4)
- Very unsupportive (5)

---

29) How encouraged did you feel to make additional comments?

- ○ Very encouraged (1)
- ○ Encouraged (2)
- ○ Neither particularly encouraged nor discouraged (3)
- ○ Discouraged (4)
- ○ Very discouraged (5)

Disclosure

For the following questions, please indicate how much you agree or disagree with statements describing how you felt during the interview.

---

30) I felt embarrassed answering these questions.

- ○ Strongly agree (1)
- ○ Somewhat agree (2)
- ○ Neither agree nor disagree (3)
- ○ Somewhat disagree (4)
- ○ Strongly disagree (5)

---

31) I felt uncomfortable answering these questions.

- Strongly agree (1)
- Somewhat agree (2)
- Neither agree nor disagree (3)
- Somewhat disagree (4)
- Strongly disagree (5)

32) Answering these questions were scary.

- Strongly agree (1)
- Somewhat agree (2)
- Neither agree nor disagree (3)
- Somewhat disagree (4)
- Strongly disagree (5)

33) I felt vulnerable answering these questions.

- Strongly agree (1)
- Somewhat agree (2)
- Neither agree nor disagree (3)
- Somewhat disagree (4)
- Strongly disagree (5)

34) I felt exposed answering these questions.

- Strongly agree (1)
- Somewhat agree (2)
- Neither agree nor disagree (3)
- Somewhat disagree (4)
- Strongly disagree (5)

---

35) I felt threatened by these questions.

- Strongly agree (1)
- Somewhat agree (2)
- Neither agree nor disagree (3)
- Somewhat disagree (4)
- Strongly disagree (5)

---

36) Answering these questions made me feel violated.

- Strongly agree (1)
- Somewhat agree (2)
- Neither agree nor disagree (3)
- Somewhat disagree (4)
- Strongly disagree (5)

---

37) I disclosed a lot in order to answer these questions truthfully.

- Strongly agree (1)
- Somewhat agree (2)
- Neither agree nor disagree (3)
- Somewhat disagree (4)
- Strongly disagree (5)

---

38) I revealed sensitive information in response to these questions.

- Strongly agree (1)
- Somewhat agree (2)
- Neither agree nor disagree (3)
- Somewhat disagree (4)
- Strongly disagree (5)

---

39) I revealed private information answering these questions.

- Strongly agree (1)
- Somewhat agree (2)
- Neither agree nor disagree (3)
- Somewhat disagree (4)
- Strongly disagree (5)

---

40) I revealed secrets in response to these questions.

- Strongly agree (1)
- Somewhat agree (2)
- Neither agree nor disagree (3)
- Somewhat disagree (4)
- Strongly disagree (5)

Perceived Anonymity

41) During the interview, I was confident that others did not know who I was.

- Strongly agree (1)
- Agree (2)
- Somewhat agree (3)
- Neither agree nor disagree (4)
- Somewhat disagree (5)
- Disagree (6)
- Strongly disagree (7)

42) During the interview, I believe that my personal identity remained unknown to others.

- Strongly agree (1)
- Agree (2)
- Somewhat agree (3)
- Neither agree nor disagree (4)
- Somewhat disagree (5)
- Disagree (6)
- Strongly disagree (7)

43) During the interview, I was easily identified as an individual by others.

- Strongly agree (1)
- Agree (2)
- Somewhat agree (3)
- Neither agree nor disagree (4)
- Somewhat disagree (5)
- Disagree (6)
- Strongly disagree (7)

44) During the interview, others were likely to know who I was.

- Strongly agree (1)
- Agree (2)
- Somewhat agree (3)
- Neither agree nor disagree (4)
- Somewhat disagree (5)
- Disagree (6)
- Strongly disagree (7)

---

45) During the interview, my personal identity was known to others.

- Strongly agree (1)
- Agree (2)
- Somewhat agree (3)
- Neither agree nor disagree (4)
- Somewhat disagree (5)
- Disagree (6)
- Strongly disagree (7)

Honesty

Please answer the following questions as truthfully as possible. **Your responses will not affect your compensation or Prolific reputation.**

---

46) During the interview, I was not as honest as I could have been.

- ○ Strongly agree (1)
- ○ Somewhat agree (2)
- ○ Neither agree nor disagree (3)
- ○ Somewhat disagree (5)
- ○ Strongly disagree (8)

---

47) I altered the details of my response when describing some of my experiences.

- ○ Yes (1)
- ○ No (3)

---

48) I withheld information when answering one or more of the questions.

- ○ Yes (1)
- ○ No (2)

---

49) Would you like to describe your responses to the previous three questions?

____

____

____

____

____

Logistics

50) During the interview, I was distracted by my surroundings.

- ◯ Strongly agree (1)
- ◯ Somewhat agree (2)
- ◯ Neither agree nor disagree (3)
- ◯ Somewhat disagree (4)
- ◯ Strongly disagree (5)

---

51) During the interview, my work environment was quiet.

- ◯ Strongly agree (1)
- ◯ Somewhat agree (2)
- ◯ Neither agree nor disagree (3)
- ◯ Somewhat disagree (4)
- ◯ Strongly disagree (5)

---

52) During the interview, I felt my conversation was not easily overheard by others.

- Strongly agree (1)
- Somewhat agree (2)
- Neither agree nor disagree (3)
- Somewhat disagree (4)
- Strongly disagree (5)

53) During the interview, I was not interrupted by others.

- Strongly agree (1)
- Somewhat agree (2)
- Neither agree nor disagree (3)
- Somewhat disagree (4)
- Strongly disagree (5)

Platform (Qualtrics)

This section was only presented to participants who completed a survey (scheduled or unscheduled) interview. Similar platform-related questions were asked to participants in other conditions.

54) Qualtrics was easy to use for the interview.

- ○ Strongly agree (1)
- ○ Somewhat agree (3)
- ○ Neither agree nor disagree (4)
- ○ Somewhat disagree (5)
- ○ Strongly disagree (7)

---

55) I felt familiar with Qualtrics, used for the interview.

- ○ Strongly agree (1)
- ○ Somewhat agree (3)
- ○ Neither agree nor disagree (4)
- ○ Somewhat disagree (5)
- ○ Strongly disagree (7)

Platform (Zoom)

This section was only presented to participants who completed a chat (anonymous or non-anonymous), audio, or video interview. Similar platform-related questions were asked to participants in other conditions.

56) Zoom was easy to use for the interview.

○ Strongly agree (1)

○ Somewhat agree (3)

○ Neither agree nor disagree (4)

○ Somewhat disagree (5)

○ Strongly disagree (7)

---

57) I felt familiar with Zoom, used for the interview.

○ Strongly agree (1)

○ Somewhat agree (3)

○ Neither agree nor disagree (4)

○ Somewhat disagree (5)

○ Strongly disagree (7)

Platform (Email)

This section was only presented to participants who completed an email interview. Similar platform-related questions were asked to participants in other conditions.

58) Email was easy to use for the interview.

- Strongly agree (1)
- Somewhat agree (3)
- Neither agree nor disagree (4)
- Somewhat disagree (5)
- Strongly disagree (7)

---

59) I felt familiar with email, used for the interview.

- Strongly agree (1)
- Somewhat agree (3)
- Neither agree nor disagree (4)
- Somewhat disagree (5)
- Strongly disagree (7)

Least and Most Favorite Question

60) During the interview, which question did you enjoy answering the most?

- ○ What are your favorite things to do in your free time? (1)
- ○ What characteristic of yourself are you most proud of? (2)
- ○ What are some of the things that make you furious? (3)
- ○ What are your attitudes about death? (4)
- ○ What are some of the things you hate about yourself? (5)
- ○ What has been the biggest disappointment in your life? (6)
- ○ What do you dislike about your physical appearance? (7)
- ○ What is your most common sexual fantasy? (8)
- ○ What have you done in your life that you feel most guilty about? (9)
- ○ What are some of the things that really hurt your feelings? (10)
- ○ What characteristics of your best friend really bother you? (11)
- ○ Can you describe the last time you were sexually aroused? (12)

61) During the interview, which question did you feel most uncomfortable answering?

- ○ What are your favorite things to do in your free time? (1)
- ○ What characteristic of yourself are you most proud of? (2)
- ○ What are some of the things that make you furious? (3)
- ○ What are your attitudes about death? (4)
- ○ What are some of the things you hate about yourself? (5)
- ○ What has been the biggest disappointment in your life? (6)
- ○ What do you dislike about your physical appearance? (7)
- ○ What is your most common sexual fantasy? (8)
- ○ What have you done in your life that you feel most guilty about? (9)
- ○ What are some of the things that really hurt your feelings? (10)
- ○ What characteristics of your best friend really bother you? (11)
- ○ Can you describe the last time you were sexually aroused? (12)

---

62) Is there anything else you'd like to share with us about your interview experience?

____

____

____

____

____

---

63 What is your Prolific ID?

____
