# Supplementary material for: Audio, video, chat, email, or survey: How much does online interview mode matter?
Source: PLoS One. 2022 Feb 22;17(2):e0263876. doi: 10.1371/journal.pone.0263876 (PMC8863281; doi:10.1371/journal.pone.0263876)
Supplement: S5 Protocol — Transcript of the post-interview survey taken by the interviewer after each interview they conducted. (PDF) [file pone.0263876.s005.pdf]

Interviewer follow-up survey

Logistics

As a reminder: Aside from completion, your answers will not affect your compensation.

1) Please provide the Prolific ID number for the corresponding interview.

____

2) Interviewer Number

- ○ 1 (1)
- ○ 2 (2)
- ○ 3 (3)
- ○ Pilot/other (4)

3) Did this interviewee attend the interview?

- ○ Yes. (1)
- ○ Was late. (2)
- ○ Cancelled at least 24 hours in advance. (3)
- ○ Cancelled less than 24 hours in advance. (4)
- ○ Was a no-show. (5)

4) How long did the interview take to complete? (Your answer will not affect your compensation.)

____

Technical Difficulties

5) Were any of the following technical difficulties encountered during the interview?

- ▢ Interviewee had difficulties joining the interview (1)
- ▢ Interviewee had difficulties using the interview platform (2)
- ▢ Poor audio quality (3)
- ▢ Poor video quality (4)
- ▢ Dropped connection (5)
- ▢ Other - please specify: (6) ____

6) How much did the technical difficulties affect the conduct of the interview?

- ○ A great deal (1)
- ○ A lot (2)
- ○ A moderate amount (3)
- ○ A little (4)
- ○ Not at all (5)

Rapport & Trust

7) How often did the respondent seem ill at ease?

- ○ During all or most of the interview (1)
- ○ At a number of points during the interview (2)
- ○ At one or two points during the interview (3)
- ○ Never (4)

8) How often did you feel ill at ease during the interview?

- ○ During all or most of the interview (1)
- ○ At a number of points during the interview (2)
- ○ At one or two points during the interview (3)
- ○ Never (4)

9) How motivated was the respondent during the interview?

- ○ Very motivated (1)
- ○ Motivated (2)
- ○ Neither particularly motivated nor unmotivated (3)
- ○ Unmotivated (4)
- ○ Very unmotivated (5)

Additional Notes

10) Did anything unusual or interesting happen during this interview? Elaborate on any responses you've given above.
