# Supplementary material for: Audio, video, chat, email, or survey: How much does online interview mode matter?
Source: PLoS One. 2022 Feb 22;17(2):e0263876. doi: 10.1371/journal.pone.0263876 (PMC8863281; doi:10.1371/journal.pone.0263876)
Supplement: S1 Table — ANOVA and Tukey comparison results testing differences in scheduling rates across mode. (PDF) [file pone.0263876.s006.pdf]

Scheduling rate by mode

ANOVA Summary

	Df	Sum Sq	Mean Sq	F value	Pr(>F)
treatment	6	3.55	0.59	2.66	0.0158
Residuals	303	67.42	0.22		

Tukey Pairwise Comparisons

	treatment.diff	treatment.lwr	treatment.upr	treatment.p.adj
Chat-Audio	-0.06	-0.34	0.23	1.00
Email-Audio	0.19	-0.11	0.49	0.52
Non-anon Chat-Audio	-0.08	-0.37	0.21	0.98
Scheduled Survey-Audio	-0.06	-0.36	0.23	0.99
Survey-Audio	0.15	-0.17	0.47	0.82
Video-Audio	-0.13	-0.41	0.14	0.78
Email-Chat	0.24	-0.06	0.54	0.21
Non-anon Chat-Chat	-0.03	-0.32	0.26	1.00
Scheduled Survey-Chat	-0.01	-0.30	0.29	1.00
Survey-Chat	0.20	-0.12	0.52	0.51
Video-Chat	-0.08	-0.35	0.20	0.98
Non-anon Chat-Email	-0.27	-0.57	0.04	0.13
Scheduled Survey-Email	-0.25	-0.56	0.06	0.21
Survey-Email	-0.04	-0.38	0.30	1.00
Video-Email	-0.32	-0.61	-0.03	0.02
Scheduled Survey-Non-anon Chat	0.02	-0.28	0.32	1.00
Survey-Non-anon Chat	0.23	-0.10	0.56	0.37
Video-Non-anon Chat	-0.05	-0.33	0.23	1.00
Survey-Scheduled Survey	0.21	-0.12	0.54	0.49
Video-Scheduled Survey	-0.07	-0.35	0.22	0.99
Video-Survey	-0.28	-0.59	0.04	0.12
