# Supplementary material for: Audio, video, chat, email, or survey: How much does online interview mode matter?
Source: PLoS One. 2022 Feb 22;17(2):e0263876. doi: 10.1371/journal.pone.0263876 (PMC8863281; doi:10.1371/journal.pone.0263876)
Supplement: S2 Table — ANOVA and Tukey comparison results testing differences in completion rates across mode. (PDF) [file pone.0263876.s007.pdf]

Completion rate by mode

ANOVA Summary

	Df	Sum Sq	Mean Sq	F value	Pr(>F)
treatment	6	6.19	1.03	4.38	0.0003
Residuals	303	71.31	0.24		

Tukey Pairwise Comparisons

	treatment.diff	treatment.lwr	treatment.upr	treatment.p.adj
Chat-Audio	0.10	-0.19	0.39	0.95
Email-Audio	0.29	-0.02	0.59	0.09
Non-anon Chat-Audio	0.09	-0.21	0.38	0.98
Scheduled Survey-Audio	0.17	-0.13	0.47	0.65
Survey-Audio	0.43	0.10	0.76	0.00
Video-Audio	-0.03	-0.32	0.25	1.00
Email-Chat	0.19	-0.12	0.50	0.55
Non-anon Chat-Chat	-0.01	-0.31	0.29	1.00
Scheduled Survey-Chat	0.07	-0.24	0.37	0.99
Survey-Chat	0.33	-0.00	0.66	0.06
Video-Chat	-0.13	-0.42	0.15	0.80
Non-anon Chat-Email	-0.20	-0.52	0.12	0.49
Scheduled Survey-Email	-0.12	-0.44	0.20	0.93
Survey-Email	0.14	-0.21	0.49	0.89
Video-Email	-0.32	-0.62	-0.02	0.03
Scheduled Survey-Non-anon Chat	0.08	-0.23	0.39	0.99
Survey-Non-anon Chat	0.34	0.00	0.68	0.05
Video-Non-anon Chat	-0.12	-0.41	0.17	0.88
Survey-Scheduled Survey	0.26	-0.08	0.60	0.27
Video-Scheduled Survey	-0.20	-0.50	0.09	0.40
Video-Survey	-0.46	-0.78	-0.14	0.00
