# Supplementary material for: Audio, video, chat, email, or survey: How much does online interview mode matter?
Source: PLoS One. 2022 Feb 22;17(2):e0263876. doi: 10.1371/journal.pone.0263876 (PMC8863281; doi:10.1371/journal.pone.0263876)
Supplement: S3 Table — ANOVA and Tukey comparison results testing differences in interviewees’ self-reported self-disclosure across mode. (PDF) [file pone.0263876.s008.pdf]

Self-reported self-disclosure by mode

ANOVA Summary

	Df	Sum Sq	Mean Sq	F value	Pr(>F)
treatment	6	4.95	0.83	1.79	0.1058
Residuals	145	66.98	0.46		

Tukey Pairwise Comparisons

	treatment.diff	treatment.lwr	treatment.upr	treatment.p.adj
Chat-Audio	-0.17	-0.82	0.48	0.99
Email-Audio	0.32	-0.32	0.97	0.74
Non-anon Chat-Audio	-0.02	-0.69	0.65	1.00
Scheduled Survey-Audio	0.31	-0.34	0.96	0.79
Survey-Audio	0.26	-0.37	0.90	0.88
Video-Audio	0.09	-0.59	0.77	1.00
Email-Chat	0.50	-0.10	1.09	0.17
Non-anon Chat-Chat	0.15	-0.47	0.77	0.99
Scheduled Survey-Chat	0.48	-0.12	1.08	0.21
Survey-Chat	0.43	-0.15	1.02	0.28
Video-Chat	0.26	-0.37	0.89	0.87
Non-anon Chat-Email	-0.34	-0.96	0.27	0.63
Scheduled Survey-Email	-0.02	-0.61	0.58	1.00
Survey-Email	-0.06	-0.64	0.51	1.00
Video-Email	-0.23	-0.86	0.39	0.92
Scheduled Survey-Non-anon Chat	0.33	-0.29	0.95	0.69
Survey-Non-anon Chat	0.28	-0.32	0.89	0.80
Video-Non-anon Chat	0.11	-0.54	0.76	1.00
Survey-Scheduled Survey	-0.05	-0.63	0.54	1.00
Video-Scheduled Survey	-0.22	-0.85	0.41	0.95
Video-Survey	-0.17	-0.79	0.44	0.98
