# Supplementary material for: Audio, video, chat, email, or survey: How much does online interview mode matter?
Source: PLoS One. 2022 Feb 22;17(2):e0263876. doi: 10.1371/journal.pone.0263876 (PMC8863281; doi:10.1371/journal.pone.0263876)
Supplement: S4 Table — ANOVA and Tukey comparison results testing differences in interviewees’ self-reported honesty across mode. (PDF) [file pone.0263876.s009.pdf]

Self-reported honesty rate by mode

ANOVA Summary

	Df	Sum Sq	Mean Sq	F value	Pr(>F)
treatment	6	2.88	0.48	0.25	0.9567
Residuals	145	272.96	1.88		

Tukey Pairwise Comparisons

	treatment.diff	treatment.lwr	treatment.upr	treatment.p.adj
Chat-Audio	0.05	-1.26	1.36	1.00
Email-Audio	-0.06	-1.36	1.24	1.00
Non-anon Chat-Audio	0.20	-1.15	1.56	1.00
Scheduled Survey-Audio	0.14	-1.18	1.45	1.00
Survey-Audio	0.31	-0.97	1.59	0.99
Video-Audio	0.30	-1.07	1.67	0.99
Email-Chat	-0.11	-1.31	1.08	1.00
Non-anon Chat-Chat	0.15	-1.10	1.41	1.00
Scheduled Survey-Chat	0.09	-1.12	1.30	1.00
Survey-Chat	0.27	-0.91	1.44	0.99
Video-Chat	0.25	-1.02	1.52	1.00
Non-anon Chat-Email	0.27	-0.98	1.51	1.00
Scheduled Survey-Email	0.20	-1.00	1.40	1.00
Survey-Email	0.38	-0.78	1.54	0.96
Video-Email	0.36	-0.90	1.62	0.98
Scheduled Survey-Non-anon Chat	-0.07	-1.32	1.19	1.00
Survey-Non-anon Chat	0.11	-1.11	1.33	1.00
Video-Non-anon Chat	0.10	-1.22	1.41	1.00
Survey-Scheduled Survey	0.18	-1.00	1.35	1.00
Video-Scheduled Survey	0.16	-1.11	1.44	1.00
Video-Survey	-0.01	-1.25	1.22	1.00
