# Supplementary material for: Audio, video, chat, email, or survey: How much does online interview mode matter?
Source: PLoS One. 2022 Feb 22;17(2):e0263876. doi: 10.1371/journal.pone.0263876 (PMC8863281; doi:10.1371/journal.pone.0263876)
Supplement: S5 Table — ANOVA and Tukey comparison results testing differences in interviewees’ self-reported perceived anonymity across mode. (PDF) [file pone.0263876.s010.pdf]

Self-reported perceived anonymity by mode

ANOVA Summary

	Df	Sum Sq	Mean Sq	F value	Pr(>F)
treatment	6	27.34	4.56	3.80	0.0015
Residuals	145	173.87	1.20		

Tukey Pairwise Comparisons

	treatment.diff	treatment.lwr	treatment.upr	treatment.p.adj
Chat-Audio	0.20	-0.84	1.25	1.00
Email-Audio	-1.11	-2.15	-0.07	0.03
Non-anon Chat-Audio	-0.51	-1.59	0.57	0.79
Scheduled Survey-Audio	-0.38	-1.43	0.67	0.93
Survey-Audio	0.05	-0.97	1.07	1.00
Video-Audio	-0.29	-1.38	0.80	0.99
Email-Chat	-1.31	-2.27	-0.36	0.00
Non-anon Chat-Chat	-0.72	-1.72	0.28	0.34
Scheduled Survey-Chat	-0.58	-1.55	0.38	0.55
Survey-Chat	-0.16	-1.09	0.78	1.00
Video-Chat	-0.49	-1.51	0.52	0.77
Non-anon Chat-Email	0.60	-0.39	1.59	0.55
Scheduled Survey-Email	0.73	-0.22	1.69	0.26
Survey-Email	1.16	0.23	2.09	0.00
Video-Email	0.82	-0.18	1.83	0.19
Scheduled Survey-Non-anon Chat	0.13	-0.87	1.14	1.00
Survey-Non-anon Chat	0.56	-0.41	1.53	0.60
Video-Non-anon Chat	0.22	-0.83	1.27	1.00
Survey-Scheduled Survey	0.43	-0.51	1.36	0.82
Video-Scheduled Survey	0.09	-0.93	1.10	1.00
Video-Survey	-0.34	-1.33	0.65	0.95
