# Supplementary material for: Audio, video, chat, email, or survey: How much does online interview mode matter?
Source: PLoS One. 2022 Feb 22;17(2):e0263876. doi: 10.1371/journal.pone.0263876 (PMC8863281; doi:10.1371/journal.pone.0263876)
Supplement: S6 Table — ANOVA and Tukey comparison results testing differences in interviewee word across mode. (PDF) [file pone.0263876.s011.pdf]

Interviewee word count by mode

ANOVA Summary

	Df	Sum Sq	Mean Sq	F value	Pr(>F)
treatment	4	12057439.05	3014359.76	10.60	0.0000
Residuals	94	26719377.50	284248.70		

Tukey Pairwise Comparisons

	treatment.diff	treatment.lwr	treatment.upr	treatment.p.adj
Chat-Audio	-754.71	-1229.78	-279.63	0.00
Email-Audio	-417.15	-872.52	38.23	0.09
Non-anon Chat-Audio	-544.81	-1039.88	-49.74	0.02
Video-Audio	206.26	-274.86	687.39	0.76
Email-Chat	337.56	-111.42	786.54	0.23
Non-anon Chat-Chat	209.89	-279.30	699.09	0.76
Video-Chat	960.97	485.90	1436.04	0.00
Non-anon Chat-Email	-127.66	-597.75	342.42	0.94
Video-Email	623.41	168.03	1078.79	0.00
Video-Non-anon Chat	751.07	256.00	1246.15	0.00
