# Supplementary material for: Audio, video, chat, email, or survey: How much does online interview mode matter?
Source: PLoS One. 2022 Feb 22;17(2):e0263876. doi: 10.1371/journal.pone.0263876 (PMC8863281; doi:10.1371/journal.pone.0263876)
Supplement: S7 Table — ANOVA and Tukey comparison results testing differences in interviewee word across mode excluding responses to followup questions. (PDF) [file pone.0263876.s012.pdf]

Interviewee word count excluding followups by mode

ANOVA Summary

	Df	Sum Sq	Mean Sq	F value	Pr(>F)
treatment	6	4461386.16	743564.36	10.01	0.0000
Residuals	142	10545622.15	74264.94		

Tukey Pairwise Comparisons

	treatment.diff	treatment.lwr	treatment.upr	treatment.p.adj
Chat-Audio	-283.99	-545.13	-22.86	0.02
Email-Audio	-95.39	-345.70	154.91	0.91
Non-anon Chat-Audio	-184.42	-456.55	87.70	0.40
Scheduled Survey-Audio	-304.85	-557.55	-52.15	0.01
Survey-Audio	-321.41	-565.50	-77.33	0.00
Video-Audio	193.53	-70.93	457.98	0.31
Email-Chat	188.60	-58.19	435.39	0.26
Non-anon Chat-Chat	99.57	-169.32	368.46	0.92
Scheduled Survey-Chat	-20.86	-270.07	228.36	1.00
Survey-Chat	-37.42	-277.89	203.06	1.00
Video-Chat	477.52	216.39	738.65	0.00
Non-anon Chat-Email	-89.03	-347.42	169.36	0.95
Scheduled Survey-Email	-209.46	-447.30	28.39	0.12
Survey-Email	-226.02	-454.69	2.65	0.05
Video-Email	288.92	38.62	539.23	0.01
Scheduled Survey-Non-anon Chat	-120.43	-381.14	140.28	0.81
Survey-Non-anon Chat	-136.99	-389.36	115.38	0.67
Video-Non-anon Chat	377.95	105.83	650.07	0.00
Survey-Scheduled Survey	-16.56	-247.85	214.73	1.00
Video-Scheduled Survey	498.38	245.68	751.08	0.00
Video-Survey	514.94	270.86	759.02	0.00
