# Supplementary material for: Audio, video, chat, email, or survey: How much does online interview mode matter?
Source: PLoS One. 2022 Feb 22;17(2):e0263876. doi: 10.1371/journal.pone.0263876 (PMC8863281; doi:10.1371/journal.pone.0263876)
Supplement: S8 Table — ANOVA and Tukey comparison results testing differences in the frequency of qualitative codes across mode. (PDF) [file pone.0263876.s013.pdf]

Qualitative code count by mode

ANOVA Summary

	Df	Sum Sq	Mean Sq	F value	Pr(>F)
treatment	4	357.53	89.38	2.37	0.0581
Residuals	94	3546.25	37.73		

Tukey Pairwise Comparisons

	treatment.diff	treatment.lwr	treatment.upr	treatment.p.adj
Chat-Audio	-4.17	-9.74	1.41	0.24
Email-Audio	-2.66	-8.07	2.76	0.65
Non-anon Chat-Audio	-4.17	-9.95	1.60	0.27
Video-Audio	0.30	-5.40	6.01	1.00
Email-Chat	1.51	-3.60	6.61	0.92
Non-anon Chat-Chat	-0.01	-5.50	5.48	1.00
Video-Chat	4.47	-0.94	9.88	0.15
Non-anon Chat-Email	-1.51	-6.84	3.81	0.93
Video-Email	2.96	-2.28	8.21	0.52
Video-Non-anon Chat	4.48	-1.14	10.10	0.18
