# Supplementary material for: Audio, video, chat, email, or survey: How much does online interview mode matter?
Source: PLoS One. 2022 Feb 22;17(2):e0263876. doi: 10.1371/journal.pone.0263876 (PMC8863281; doi:10.1371/journal.pone.0263876)
Supplement: S9 Table — ANOVA and Tukey comparison results testing differences in the frequency of qualitative codes across mode excluding responses to followup questions. (PDF) [file pone.0263876.s014.pdf]

Qualitative code count excluding followups by mode

ANOVA Summary

	Df	Sum Sq	Mean Sq	F value	Pr(>F)
treatment	6	266.03	44.34	1.58	0.1567
Residuals	140	3923.58	28.03		

Tukey Pairwise Comparisons

	treatment.diff	treatment.lwr	treatment.upr	treatment.p.adj
Chat-Audio	-1.27	-6.44	3.89	0.99
Email-Audio	1.68	-3.34	6.70	0.95
Non-anon Chat-Audio	-0.50	-5.85	4.86	1.00
Scheduled Survey-Audio	-1.55	-6.62	3.52	0.97
Survey-Audio	-1.54	-6.52	3.44	0.97
Video-Audio	1.69	-3.60	6.98	0.96
Email-Chat	2.96	-1.77	7.69	0.50
Non-anon Chat-Chat	0.78	-4.31	5.86	1.00
Scheduled Survey-Chat	-0.28	-5.06	4.50	1.00
Survey-Chat	-0.27	-4.95	4.42	1.00
Video-Chat	2.96	-2.05	7.98	0.57
Non-anon Chat-Email	-2.18	-7.12	2.76	0.84
Scheduled Survey-Email	-3.23	-7.86	1.39	0.36
Survey-Email	-3.22	-7.75	1.30	0.34
Video-Email	0.01	-4.86	4.87	1.00
Scheduled Survey-Non-anon Chat	-1.05	-6.04	3.93	1.00
Survey-Non-anon Chat	-1.04	-5.94	3.85	1.00
Video-Non-anon Chat	2.19	-3.02	7.40	0.87
Survey-Scheduled Survey	0.01	-4.57	4.58	1.00
Video-Scheduled Survey	3.24	-1.67	8.15	0.44
Video-Survey	3.23	-1.59	8.05	0.42
