# Supplementary material for: Audio, video, chat, email, or survey: How much does online interview mode matter?
Source: PLoS One. 2022 Feb 22;17(2):e0263876. doi: 10.1371/journal.pone.0263876 (PMC8863281; doi:10.1371/journal.pone.0263876)
Supplement: S11 Table — ANOVA and Tukey comparison results testing differences in the frequency of rare qualitative codes (two standard deviations method) across mode excluding responses to followup questions. (PDF) [file pone.0263876.s016.pdf]

Rare qualitative code count excluding followups by mode (standard deviation)

ANOVA Summary

	Df	Sum Sq	Mean Sq	F value	Pr(>F)
treatment	6	0.37	0.06	1.62	0.1464
Residuals	140	5.38	0.04		

Tukey Pairwise Comparisons

	treatment.diff	treatment.lwr	treatment.upr	treatment.p.adj
Chat-Audio	0.05	-0.14	0.24	0.99
Email-Audio	0.00	-0.19	0.19	1.00
Non-anon Chat-Audio	-0.00	-0.20	0.20	1.00
Scheduled Survey-Audio	-0.00	-0.19	0.19	1.00
Survey-Audio	0.12	-0.06	0.30	0.45
Video-Audio	0.11	-0.09	0.30	0.68
Email-Chat	-0.05	-0.22	0.13	0.98
Non-anon Chat-Chat	-0.05	-0.24	0.14	0.99
Scheduled Survey-Chat	-0.05	-0.22	0.13	0.98
Survey-Chat	0.07	-0.10	0.25	0.87
Video-Chat	0.06	-0.13	0.24	0.97
Non-anon Chat-Email	-0.00	-0.18	0.18	1.00
Scheduled Survey-Email	-0.00	-0.17	0.17	1.00
Survey-Email	0.12	-0.05	0.29	0.33
Video-Email	0.11	-0.07	0.29	0.58
Scheduled Survey-Non-anon Chat	-0.00	-0.18	0.18	1.00
Survey-Non-anon Chat	0.12	-0.06	0.30	0.43
Video-Non-anon Chat	0.11	-0.09	0.30	0.66
Survey-Scheduled Survey	0.12	-0.05	0.29	0.35
Video-Scheduled Survey	0.11	-0.08	0.29	0.60
Video-Survey	-0.01	-0.19	0.16	1.00
