# Supplementary material for: Audio, video, chat, email, or survey: How much does online interview mode matter?
Source: PLoS One. 2022 Feb 22;17(2):e0263876. doi: 10.1371/journal.pone.0263876 (PMC8863281; doi:10.1371/journal.pone.0263876)
Supplement: S12 Table — ANOVA and Tukey comparison results testing differences in the frequency of rare qualitative codes (first quartile method) across mode. (PDF) [file pone.0263876.s017.pdf]

Rare qualitative code count by mode (quartiles)

ANOVA Summary

	Df	Sum Sq	Mean Sq	F value	Pr(>F)
treatment	4	22.50	5.62	4.44	0.0025
Residuals	94	119.14	1.27		

Tukey Pairwise Comparisons

	treatment.diff	treatment.lwr	treatment.upr	treatment.p.adj
Chat-Audio	0.08	-0.18	0.35	0.90
Email-Audio	0.02	-0.23	0.28	1.00
Non-anon Chat-Audio	-0.06	-0.33	0.21	0.97
Video-Audio	0.10	-0.17	0.37	0.84
Email-Chat	-0.06	-0.30	0.18	0.96
Non-anon Chat-Chat	-0.14	-0.40	0.12	0.54
Video-Chat	0.02	-0.24	0.27	1.00
Non-anon Chat-Email	-0.08	-0.33	0.17	0.89
Video-Email	0.07	-0.17	0.32	0.92
Video-Non-anon Chat	0.16	-0.11	0.42	0.46
