# Supplementary material for: Audio, video, chat, email, or survey: How much does online interview mode matter?
Source: PLoS One. 2022 Feb 22;17(2):e0263876. doi: 10.1371/journal.pone.0263876 (PMC8863281; doi:10.1371/journal.pone.0263876)
Supplement: S13 Table — ANOVA and Tukey comparison results testing differences in the frequency of rare qualitative codes (first quartile method) across mode excluding responses to followup questions. (PDF) [file pone.0263876.s018.pdf]

Rare qualitative code count excluding followups by mode (quartiles)

ANOVA Summary

	Df	Sum Sq	Mean Sq	F value	Pr(>F)
treatment	6	8.96	1.49	2.10	0.0572
Residuals	140	99.70	0.71		

Tukey Pairwise Comparisons

	treatment.diff	treatment.lwr	treatment.upr	treatment.p.adj
Chat-Audio	-0.84	-1.67	-0.02	0.04
Email-Audio	-0.63	-1.44	0.17	0.22
Non-anon Chat-Audio	-0.62	-1.47	0.23	0.32
Scheduled Survey-Audio	-0.52	-1.33	0.28	0.46
Survey-Audio	-0.58	-1.37	0.22	0.32
Video-Audio	-0.23	-1.07	0.61	0.98
Email-Chat	0.21	-0.55	0.96	0.98
Non-anon Chat-Chat	0.22	-0.59	1.03	0.98
Scheduled Survey-Chat	0.32	-0.44	1.08	0.87
Survey-Chat	0.27	-0.48	1.01	0.94
Video-Chat	0.61	-0.19	1.41	0.25
Non-anon Chat-Email	0.01	-0.77	0.80	1.00
Scheduled Survey-Email	0.11	-0.63	0.85	1.00
Survey-Email	0.06	-0.66	0.78	1.00
Video-Email	0.41	-0.37	1.18	0.70
Scheduled Survey-Non-anon Chat	0.10	-0.70	0.89	1.00
Survey-Non-anon Chat	0.04	-0.74	0.82	1.00
Video-Non-anon Chat	0.39	-0.44	1.22	0.79
Survey-Scheduled Survey	-0.05	-0.78	0.68	1.00
Video-Scheduled Survey	0.30	-0.49	1.08	0.92
Video-Survey	0.35	-0.42	1.12	0.83
