# Supplementary material for: Audio, video, chat, email, or survey: How much does online interview mode matter?
Source: PLoS One. 2022 Feb 22;17(2):e0263876. doi: 10.1371/journal.pone.0263876 (PMC8863281; doi:10.1371/journal.pone.0263876)
Supplement: S14 Table — ANOVA and Tukey comparison results testing differences in the frequency of rare qualitative codes (first quartile method) across mode. One outlier was removed to test its effect. (PDF) [file pone.0263876.s019.pdf]

Rare qualitative code count by mode with outlier dropped (quartiles)

ANOVA Summary

	Df	Sum Sq	Mean Sq	F value	Pr(>F)
treatment	4	15.35	3.84	3.51	0.0102
Residuals	93	101.64	1.09		

Tukey Pairwise Comparisons

	treatment.diff	treatment.lwr	treatment.upr	treatment.p.adj
Chat-Audio	-1.02	-1.99	-0.06	0.03
Email-Audio	-1.02	-1.96	-0.08	0.03
Non-anon Chat-Audio	-0.80	-1.80	0.20	0.18
Video-Audio	-0.32	-1.31	0.67	0.90
Email-Chat	0.00	-0.87	0.87	1.00
Non-anon Chat-Chat	0.22	-0.71	1.16	0.96
Video-Chat	0.70	-0.22	1.62	0.22
Non-anon Chat-Email	0.22	-0.68	1.13	0.96
Video-Email	0.70	-0.19	1.59	0.19
Video-Non-anon Chat	0.48	-0.48	1.44	0.63
