# Supplementary material for: Audio, video, chat, email, or survey: How much does online interview mode matter?
Source: PLoS One. 2022 Feb 22;17(2):e0263876. doi: 10.1371/journal.pone.0263876 (PMC8863281; doi:10.1371/journal.pone.0263876)
Supplement: S15 Table — ANOVA and Tukey comparison results testing differences in the frequency of rare qualitative codes (first quartile method) across mode excluding responses to followup questions. One outlier was removed to test its effect. (PDF) [file pone.0263876.s020.pdf]

Rare qualitative code count excluding followups by mode with outlier dropped (quartiles)

ANOVA Summary

	Df	Sum Sq	Mean Sq	F value	Pr(>F)
treatment	6	7.01	1.17	1.69	0.1279
Residuals	139	96.17	0.69		

Tukey Pairwise Comparisons

	treatment.diff	treatment.lwr	treatment.upr	treatment.p.adj
Chat-Audio	-0.73	-1.56	0.10	0.12
Email-Audio	-0.52	-1.32	0.28	0.46
Non-anon Chat-Audio	-0.51	-1.36	0.35	0.57
Scheduled Survey-Audio	-0.41	-1.22	0.40	0.74
Survey-Audio	-0.46	-1.26	0.33	0.59
Video-Audio	-0.12	-0.96	0.73	1.00
Email-Chat	0.21	-0.54	0.95	0.98
Non-anon Chat-Chat	0.22	-0.58	1.02	0.98
Scheduled Survey-Chat	0.32	-0.43	1.07	0.86
Survey-Chat	0.27	-0.47	1.00	0.93
Video-Chat	0.61	-0.17	1.40	0.24
Non-anon Chat-Email	0.01	-0.76	0.79	1.00
Scheduled Survey-Email	0.11	-0.62	0.84	1.00
Survey-Email	0.06	-0.65	0.77	1.00
Video-Email	0.41	-0.36	1.17	0.69
Scheduled Survey-Non-anon Chat	0.10	-0.69	0.88	1.00
Survey-Non-anon Chat	0.04	-0.72	0.81	1.00
Video-Non-anon Chat	0.39	-0.43	1.21	0.78
Survey-Scheduled Survey	-0.05	-0.77	0.67	1.00
Video-Scheduled Survey	0.30	-0.48	1.07	0.91
Video-Survey	0.35	-0.41	1.10	0.82
