# Supplementary material for: Audio, video, chat, email, or survey: How much does online interview mode matter?
Source: PLoS One. 2022 Feb 22;17(2):e0263876. doi: 10.1371/journal.pone.0263876 (PMC8863281; doi:10.1371/journal.pone.0263876)
Supplement: S16 Table — Multiple t-test results comparing screening survey results pre-pandemic and during the pandemic including the availability of remote interview resources, accessibility of a private space, and willingness to participate in various interview modes. (PDF) [file pone.0263876.s021.pdf]

Effect of the Covid-19 Pandemic

To compare proportions before and after the pandemic, we completed a series of two-sample t-tests with equal variances assumed. There are small differences in n across tests because the survey questions were optional and some participants chose not to answer.

Access to a webcam

Table 1. Webcam access pre/post pandemic

Statistic	Value
t-stat	-1.1774
p	0.239
pre-pandemic n	1238
post-pandemic n	100

Access to a quiet, private room

Table 2. Private room access pre/post pandemic

Statistic	Value
t-stat	1.2453
p	0.213
pre-pandemic n	1237
post-pandemic n	100

Willingness to be recorded

Table 3. Willingness to be recorded pre/post pandemic

Statistic	Value
t-stat	-0.7299
p	0.466
pre-pandemic n	1234
post-pandemic n	100

Willingness to participate in a survey interview

Table 4. Willingness to participate in a survey interview pre/post pandemic

Statistic	Value
t-stat	1.6611
p	0.097
pre-pandemic n	1240
post-pandemic n	100

Willingness to participate in a chat interview

Table 5. Willingness to participate in a chat interview pre/post pandemic

Statistic	Value
t-stat	1.4736
p	0.141
pre-pandemic n	1240
post-pandemic n	100

Willingness to participate in an email interview

Table 6. Willingness to participate in an email interview pre/post pandemic

Statistic	Value
t-stat	1.888
p	0.059
pre-pandemic n	1240
post-pandemic n	100

Willingness to participate in an audio interview

Table 7. Willingness to participate in an audio interview pre/post pandemic

Statistic	Value
t-stat	1.2357
p	0.217
pre-pandemic n	1240
post-pandemic n	100

Willingness to participate in a video interview

Table 8. Willingness to participate in a video interview pre/post pandemic

Statistic	Value
t-stat	0.7805
p	0.435
pre-pandemic n	1240
post-pandemic n	100
