# Supplementary material for: Audio, video, chat, email, or survey: How much does online interview mode matter?
Source: PLoS One. 2022 Feb 22;17(2):e0263876. doi: 10.1371/journal.pone.0263876 (PMC8863281; doi:10.1371/journal.pone.0263876)
Supplement: Attachment — Submitted filename: PLOS ONE Response to Comments II.pdf [file pone.0263876.s022.pdf]

We appreciate the reviewers' constructive feedback and have incorporated changes based on their comments into the revised version of the manuscript. Our response is organized as follows: first we cover the most significant comments that were primarily related to the interpretation of results, then we move on to address the issues raised regarding our methodology, and finally we conclude with comments related to potential bias stemming from the study design. In addition to the comments addressed below, several minor revisions were made to address grammatical errors, issues with direct quotes, and a discrepancy in the total number of interviews reported (154) noted by the reviewers.

Comments Related to Discussion and Interpretation

Comment (Reviewer 1): *The authors' first conclusion that mode is only a practical question, and not a threat to validity, is not supported by relevant literature on modes and accuracy of response and also not supported by two of their findings. First, as the authors note, the greatest challenge to recruitment (and logistics) involved audio and video mode. Given that 99% of screened participants indicated having access to a computer with a keyboard and 70% having a webcam, it seems like unwillingness to participate in audio or video modes was influenced (at least in part) by recruits' lack of comfort with reporting in those modes. Audio and video do complicate logistics, as the authors note, but having these devices suggests these participants should be comfortable using them. Even though the authors did not find significant results across modes in their analysis of disclosure, they note on page 36 that interviewers might overestimate participants' comfort and on page 42 that some participants indicated mode affected their disclosure. So, this first conclusion seems stronger than the research demonstrates, especially considering the noted limitation in sample size that affected all quantitative analysis.*

Response: To start with the most considerable modification, we agree with Reviewer 1 that our first conclusion regarding mode as a practical question was too strong and required further qualification. While the primary differences observed across mode were related to logistics, and these differences have practical implications for researchers, we agree that mode is not only a practical decision and there are valid concerns about bias and validity pertaining to mode. As such, we reframed this section to discuss the practical considerations that mode raises as well as potential bias related to self-selection (where more personally revealing modes may dissuade shy, self-conscious, or private individuals) and systematic exclusion (where lack of access to a private space or a good internet connection might preclude participation). Therefore, we modified our conclusions to (1) highlight the lack of differences in the structure and thematic content of the data collected across mode, (2) identify the logistical challenges related to practicality and validity, and (3) discuss other contextual factors that are important when deciding upon interview mode.

Comment (Reviewer 2): *Differences between qualitative methodologies is not addressed. For instance, a phenomenological study would not be amenable to data collection via a survey, nor would coding of data be a consideration. Ethnographic studies also typically rely on observational data, and this is not mentioned at all. You duly acknowledge that there is no universal approach to qualitative data analysis,*

however you have not stated that the approach depends on the aims of the research and the methodology (consider narrative analysis, discourse analysis, etc.). It seems that the findings of the study are applicable to mixed methods research, but not so much to purely qualitative research (although this clearly depends on the methodology). Perhaps this could be considered and discussed.

Response: As a part of the contextual elements that influence the choice of interview mode (point 3 from above), we agree with Reviewer 2 that we were missing a discussion of how the qualitative methodology and underlying aim of the research affect the decision. We incorporated this aspect into the discussion section and further restricted the applicability of our results to qualitative interview studies specifically rather than qualitative research more broadly.

Comment (Reviewer 1): *On page 27, the authors note that 99% of screened participants indicated having access to a computer with a keyboard. Part c. of Table 4 (on page 26) also shows participants were disproportionately white. Like most examples of online research I have seen, this sample is clearly skewed to higher average SES and White participants. Although it seems likely that the authors could not have obtained a more representative sample without significantly changing recruitment methods, it would be useful for the authors to comment on how this skew in participant demographics might have affected results across all experimental conditions?*

Response: As a part of the above changes, we also addressed two of Reviewer 1's comments related to bias in our recruited sample. First, as Reviewer 1 correctly notes, our sample overrepresents white participants and, while we did not collect socioeconomic data directly, exhibits a skew associated with higher income. When reporting our sample's demographics, we added a discussion of this overarching bias in the sample and in our discussion we limited the generalizability of our results to samples recruited through online panels which are likely to encounter similar bias. In addition, we discuss how access to technology, private space, and a good internet connection, all larger hurdles for individuals with lower income, can affect both recruitment in online studies and a researcher's decision to use a certain mode. However, we also note that while we observe this general skew in race and other socioeconomic indicators, these factors were distributed relatively equally across mode and therefore are unlikely to have affected our comparison of results between conditions.

Comment (Reviewer 1): *The authors note on page 24 that the greatest challenges to recruitment and logistics involved audio and video modes. They specifically note on page 26 that unwillingness to participate in video mode was the greatest disqualifying factor in the recruited pool. This is not surprising, but good to note for practitioners who are considering different online modes. A separate question is how the lower screening and completion rates and the higher no-show rates for audio and video modes (as reported in Table 5 on page 32). How might this differential nonresponse pattern have affected findings and conclusions?*

Response: As for Reviewer 1's second comment regarding bias, we agree that differences in eligibility, completion, and no-show rates for audio and video do affect our conclusions. As previously discussed, we added several sections to the findings and discussion sections that highlight the potential self-selection bias that these modes introduce. We reason that while users may be comfortable with the

process of using a device, they may not be comfortable with having an interviewer see their face or their surroundings, hear their voice, or perhaps be overheard by others who live or work in their space. Thus discomfort is not limited to knowledge of using a device. Furthermore, participants may not be interested in participating in a study with video or audio because it would require them to participate in a quiet space with a good internet connection and may be less conducive to multi-tasking.

Comments Related to the Methodology

Comment (Reviewer 2): *You have presented a very well-written and clear manuscript. You aptly highlighted the need for the novel research and detailed the methods, and findings meticulously. Although I thoroughly enjoyed reading it for its clarity and novelty, I must admit that it was challenging for me at times as a qualitative researcher. The statement on page 11, line 281 ("we were wary of attempting to assess a qualitative method using quantitative …"), was a reassuring touch.*

Response: In terms of our methods, we certainly agree with the skepticism expressed by Reviewer 2 and appreciate their acknowledgement of our similar hesitancy to apply qualitative analysis to evaluate quantitative research methods. This was part of the reason why we chose to move our discussion of the limitations and validity of our study further up in the paper (in the methods section) rather than the more typical location as a part of the discussion which Reviewer 2 noted. In doing so, we hoped to express these considerations up front and provide additional context before presenting our findings.

Comment (Reviewer 2): *I was surprised to see you refer to the limitations in the method section, however, I recognise this may be a more accepted convention in quantitative research papers.*

Response: As discussed above, our aim in moving the limitations into the methods section, rather than the discussion, was to highlight limitations and the context in which our results should be interpreted before diving into them.

Comment (Reviewer 2): *On page 48, code count is discussed as a measure of thematic analysis potential. You acknowledge that this is simplistic, a point with which I agree, but I also challenge this as a meaningful measure of the qualitative equivalence. To my mind, the number of codes (i.e., ways to break up and categorise data) does not speak to its interpretative potential. At minimum, a rationale for including this as a measure with an appropriate reference would be useful.*

Response: We also agree with Reviewer 2 that code count is not a strong indicator of thematic analysis potential and restricted our interpretation to what it should have been to start: a proxy for the breadth of content disclosed during the interview. In the methods section we added our reasoning for using this outcome measure, namely that the ``generation of themes via coding... and categorization... is arguably the most common analytic approach taken by qualitative researchers using interviews'' (Roulston 2014 in SAGE Handbook of Qualitative Data Analysis). As a common methodology in qualitative research, we

propose that differences in the frequency and distribution of codes across mode would be more insightful than assessing differences in word count alone.

Comment (Reviewer 2): *It would be useful to know if any qualitative data analysis software or tools was used by the coders.*

Response: Related to the qualitative coding, we added clarification to the methods section to specify that the qualitative coding did not use any specific qualitative research software, only shared spreadsheet templates created by the research team.

Comment (Reviewer 2): *The study title and aims suggest that the research compares empirically, different modes of online interviews, and yet you have included surveys. You note that surveys are "the interview's close cousins" however, qualitative researchers would likely argue that they are a different data collection technique to interviews altogether (i.e., rather than a mode of online interview). The rationale for including survey techniques in the study is not overly satisfying and it seems that more needs to be said about the apparent juxtaposition of online interview modes and survey techniques.*

Response: We agree with Reviewer 2 that surveys and interviews are inherently distinct methodologies. While we treated the two very differently in our quantitative analysis, having two sets of statistical tests (one with surveys and one without) for most outcome measures, we did not present the two as such in our introduction, related work, or methods sections. As such, we modified our presentation of the two methods to highlight their differences, specifically removing the sentence the reviewer referred to in their comment. In addition, we added further justification for our inclusion of the survey conditions which were to be used as a baseline for comparison and as a means of evaluating the effects of scheduling-- something that would have been very difficult and expensive to do using interview methods alone.

Comments Related to Potential Bias

Comment (Reviewer 1): *For example, an analysis of interviewer effects showing interviewers' contribution to variance in participants responses to questions. The authors note on page 16 that they examined interview completion rates, mode completion rates, technical difficulties, and rapport rating and did not see any differences. These are useful metrics to report, but do not actually identify interview effects. If the authors cannot estimate interviewers' contribution to variance due to sample size or study design, are there any further indirect metrics that could inform the likely potential for interviewer effects?*

Response: Reviewer 1 asked about additional metrics that might be able to better identify potential interviewer effects. We agree that measuring interviewer effects directly is difficult and had attempted to minimize this possibility using three interviewers with similar traits: "all three were white, women, aged 20-25 years, fluent English-speakers, willing to broach sensitive topics, with a similar gender presentation and hairstyles, and who had no previous experience with research interviewing" (page 15).

In addition to evidence that completion rates, technical difficulties, and rapport rating did not vary by interviewer, we also added that the honesty self-report, word count, and qualitative code count did not substantially vary by interviewer either.

Comment (Reviewer 1): *The main questions the authors test are clearly designed to represent questions with different degrees of sensitivity. These questions do not seem like questions that would be asked by survey or market researchers conducting online interviews. Including some "standard" survey questions would have provided readers with more confidence that these results are relevant to their own research. Can the authors comment on how their selection of questions could have affected the results?*

Response: Reviewer 1 noted that the choice of interview questions, specifically their sensitive nature, might provide different results than standard survey or interview questions. We agree with this point as it was an explicit design decision. According to Nandi and Platt (2017), differences in self-disclosure, perceived anonymity, and honesty should be more pronounced in interviews focusing on sensitive topics (discussed on page 9 and 14). Therefore, we reasoned that if we were to find differences in mode it would be in these kinds of sensitive interviews and accordingly we would expect to see an even smaller effect in interviews with less sensitive and more standard content. We elaborated on this in the methods section as well as made it a specific point to probe in the discussion section.

Comment (Reviewer 1): *The interviewee post-interview survey was a useful design feature, but responses to some of these questions - desirable responding, self-disclosure, and honesty - could have been directly influenced by the specific questions in the initial interview.*

Response: Reviewer 1 raised concern that the choice of interview questions might bias the responses in the post-interview survey. We agree that measures that the reviewer identifies (social desirability, honesty, and self-disclosure) could be affected by the sensitive nature of the interview questions. However, since (1) our aim was to identify differences across mode, (2) the questions were the same across all conditions, and (3) we would not expect the questions to have a differential effect across mode, we do not believe this affected our results.
