# Supplementary material for: Audio, video, chat, email, or survey: How much does online interview mode matter?
Source: PLoS One. 2022 Feb 22;17(2):e0263876. doi: 10.1371/journal.pone.0263876 (PMC8863281; doi:10.1371/journal.pone.0263876)
Supplement: Attachment — Submitted filename: PLOS ONE Response To Comments - Revision II.pdf [file pone.0263876.s023.pdf]

Thank you to the reviewers for taking the time to provide additional comments and to clarify some of the points that we had not fully addressed, or had misunderstood, in the initial revision. Below we respond to each of the reviewer's comments individually and include a summary of the changes we made in the manuscript to address each point.

Comment (Reviewer 1): *In response to my comment about how the observed skew in participant demographics to white and higher SES participants might have affected results (across all experimental conditions, the authors note that these factors were distributed relatively equally across mode and therefore are unlikely to have affected our comparison of results between conditions. The authors' then conclude that this demographic skew should not have affected the internal validity of their experimental study, given the similar composition across conditions. To clarify my original question, a number of the authors' conclusions include claims of external validity – that is, what other researchers should expect in terms of data quality when using different modes of online interviewing. Can the authors address the issue of how the demographic skew of their study participants could have affected the ability of their study to provide external validity?*

Response: We realize that we had misinterpreted the reviewer's initial comments and agree that the demographic skew in our data does pose a challenge to external validity that we had not adequately acknowledged in the paper. We made two changes accordingly. First, we added several lines to the "Participant demographics" sub-section (lines 708-719 in the tracked changes document) that explore the differences between interviews with black participants and those with Latino and white participants. We use this data to indicate that there are possible differences that should make researchers cautious in applying our findings, but we also state that we cannot draw specific conclusions due to the low number of black and Latino participants in each condition (about 1-2 participants in each group per arm). This prevented any meaningful subgroup analysis. Second, we expanded on our discussion of socioeconomic skew and disproportionate access to technology in the following section (lines 755-759 in the tracked changes document) to be explicit about challenges to internal versus external validity. We discuss how our sample is skewed from the general population but also note that it is a suboptimal reality that, by the same token, our sample is likely representative of online research panels. Therefore, we conclude that researchers using similar methods of recruitment can likely apply our findings directly but other researchers, particularly those working with low-income or marginalized groups, should be tentative in drawing on the same conclusions.

Comment (Reviewer 1): *The authors note on page 51 that "despite low power, any effects on interview data that was missed in this study are likely small enough that most interviewers can gently ignore them." First, I assume the authors meant to say "generally" instead of gently. Second, this statement feels a bit strong, given that the authors have no statistical criteria to determine whether observed effects should, or should not, be considered meaningful. The authors' conclusion might be correct (and I would guess it is, based on their results), but assuming this is the case without statistical support or other metrics seems questionable. Do the authors have other evidence or literature they can cite to support this conclusion that the effects are likely small enough to be ignorable?*

Response: Our original intention in using the word "gently" was to convey that the effects could be ignored with caution but shouldn't be ignored completely. However, we agree that the word "gently" is confusing so we changed it to use the word "cautiously" to make this point more clearly. We, presumably like the reviewer, feel that "generally" would make this statement too strong and in general felt that the language we use should be softened. It now reads "Thus despite low power, the effects of mode on interview data are likely small enough that most interviewers can cautiously ignore them." In addition to the wording, we felt that the reviewer's criticism regarding the lack of supporting evidence in this section was a fair point. We believe that our statistically insignificant results across most of our primary metrics justify the intent behind our claim, but to make a stronger case we expanded upon our findings in this section (lines 1412-1424 in the tracked changes document). Specifically we point to the small to moderate effect sizes that we observe in the data measured using Cohon's d (with citation) and discuss how larger sample sizes, while possibly making these effects statistically significant, would only decrease the relative effect size.

Comment (Reviewer 1): *Further in the same paragraph, the authors conclude that they "... expect the effect of mode to be even smaller for interviews that focus on less sensitive content." This statement makes intuitive sense to me but given that the authors (intentionally) did not include less sensitive questions in the experimental design or cite relevant literature here, the basis of this statement needs to be more clearly justified.*

Response: In the referenced paragraph we were missing the relevant citation from Nandi and Platt (2017) that had been used in a similar discussion of sensitive interview questions in the related works section. We reworded this sentence to make it clear that the justification for this claim draws from previous work rather than our own findings. It now reads "Furthermore, evidence from previous work suggests that the effect of mode is likely even smaller for interviews that focus on less sensitive content [36]". In reviewing this claim, we felt that an explicit reference and discussion should also be included in the related works sub-section entitled "Sensitive content" so several lines were added there (lines 157-161 in the tracked changes document).

Comment (Reviewer 2): *You have gone some way in addressing the key criticism made about methodology versus data collection method however, I do still feel that there is some conflation of qualitative research and interviews. Specifically, on p. 5, under the sub-heading "Honesty", you state, "In qualitative research, the honesty of a participant's response is a fundamental underpinning of the field." I would argue this is also the case in quantitative research, where self-report measures are used (e.g., validated pain scales, perceived knowledge of a particular topic, satisfaction surveys, etc.). Equally, ethnographic studies, for example, data is collected in part by researchers' observations of participants (no honesty required on participants' part). I feel that some further clarity (a sentence or two) about the fact that you are not investigating qualitative research methods generally, but rather a data collection method commonly used in qualitative research, namely interviews.*

Response: We appreciate that the reviewer acknowledged our changes in response to their original comments and agree that a few further changes are necessary. First, in the referenced section, the reviewer is correct that honesty is an important underpinning of both qualitative and quantitative research, particularly those that rely on self-reported measures. Therefore, we shifted the start of the discussion in the “Honesty” sub-section to focus on human subjects research rather than qualitative methods specifically (lines 117-122 in the tracked changes document). In particular we acknowledge that both qualitative and quantitative researchers in this area face the challenge of accurately capturing how people behave and think in the real world. Therefore, honesty in self-reported measures are equally important, and challenging, in both contexts. Second, to clearly distinguish between research on interview methods (our work) and that on qualitative research methods, we added a paragraph to the end of the introduction where we introduce our study and its contributions (lines 64-71 in the tracked changes document). In this section we explicitly state that our research focuses on online interviews as a data collection method, the interpretation of our findings are limited to that context, and we do not attempt to expand our conclusions beyond that to qualitative methods more broadly.

Comment (Reviewer 2): *There are some other minor points for your consideration on p. 37 (lines 965-966) the terms interviewee and participant are used interchangeably which is a bit confusing.*

Response: We changed the referenced section to use the term “interviewee” exclusively to avoid confusion. We felt like this point warranted further examination beyond just this section so we changed “participant” to “interviewee” in several other findings sections where it made the discussion clearer. In general, we standardized the terminology to use “interviewee” when discussing findings pertaining to the content of the interviews or self-reported measures from the surveys. Special attention was paid to cases where there was a discussion of both interviewees’ and interviewers’ experiences in the same section.

Comment (Reviewer 2): *There is a minor typographic error on p.7 (line 157) “interviewees”.*

Response: This was changed from “Social desirability bias is a phenomenon where interviewee’s…” to “Social desirability bias is a phenomenon where interviewees…”
